# Reduction of SPARC protects mice against NLRP3 inflammasome activation and obesity

**DOI:** 10.1172/JCI169173

**Published:** 2023-10-02

**Authors:** Seungjin Ryu, Olga Spadaro, Sviatoslav Sidorov, Aileen H. Lee, Sonia Caprio, Christopher Morrison, Steven R. Smith, Eric Ravussin, Irina Shchukina, Maxim N. Artyomov, Yun-Hee Youm, Vishwa Deep Dixit

**Affiliations:** 1 Department of Pathology and; 2 Department of Immunobiology, Yale School of Medicine, New Haven, Connecticut, USA.; 3 Department of Pharmacology, College of Medicine, Hallym University, Chuncheon, Gangwon, South Korea.; 4 Department of Pediatrics, Yale School of Medicine, New Haven, Connecticut, USA.; 5 Pennington Biomedical Research Center, Baton Rouge, Louisiana, USA.; 6 Translational Research Institute for Metabolism and Diabetes, AdventHealth, Orlando, Florida, USA.; 7 Department of Pathology and Immunology, Washington University School of Medicine, St. Louis, Missouri, USA.; 8 Yale Center for Research on Aging, Yale School of Medicine, New Haven, Connecticut, USA.

**Keywords:** Inflammation, Metabolism, Adipose tissue, Innate immunity, Obesity

## Abstract

The comprehensive assessment of long-term effects of reducing intake of energy (CALERIE-II; NCT00427193) clinical trial established that caloric restriction (CR) in humans lowers inflammation. The identity and mechanism of endogenous CR-mimetics that can be deployed to control obesity-associated inflammation and diseases are not well understood. Our studies have found that 2 years of 14% sustained CR in humans inhibits the expression of the matricellular protein, secreted protein acidic and rich in cysteine (SPARC), in adipose tissue. In mice, adipose tissue remodeling caused by weight loss through CR and low-protein diet feeding decreased, while high-fat diet–induced (HFD-induced) obesity increased SPARC expression in adipose tissue. Inducible SPARC downregulation in adult mice mimicked CR’s effects on lowering adiposity by regulating energy expenditure. Deletion of SPARC in adipocytes was sufficient to protect mice against HFD-induced adiposity, chronic inflammation, and metabolic dysfunction. Mechanistically, SPARC activates the NLRP3 inflammasome at the priming step and downregulation of SPARC lowers macrophage inflammation in adipose tissue, while excess SPARC activated macrophages via JNK signaling. Collectively, reduction of adipocyte-derived SPARC confers CR-like metabolic and antiinflammatory benefits in obesity by serving as an immunometabolic checkpoint of inflammation.

## Graphical abstract

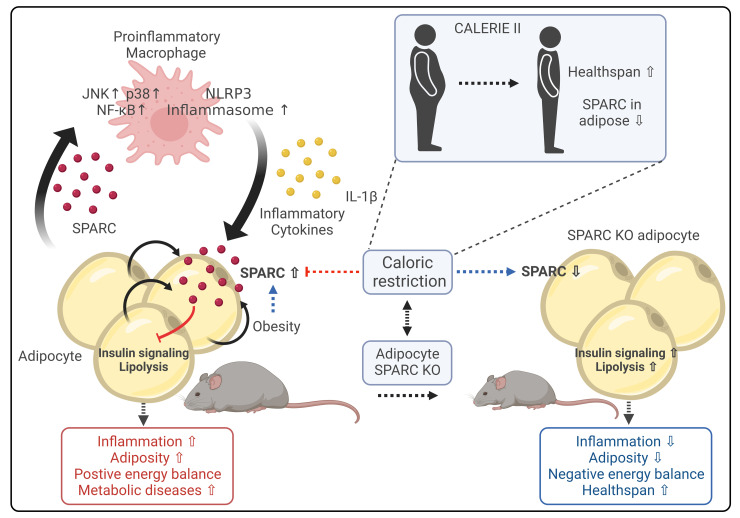

## Introduction

Substantial research effort has been invested to identify the caloric restriction (CR) mimetics that can be deployed to enhance lifespan and health span (1). However, the longevity dividend produced by 40% CR in laboratory animals is associated with tradeoffs that suppress organismal growth, reproduction, and immune-defense (2–8). Importantly, the inability to anticipate temporary lack of nutrients through forced restriction of calories in nonconsenting animals may also elicit an evolutionarily conserved stress response evidenced by increased glucocorticoids (9), which can further obfuscate the identification of endogenous drivers of CR’s effect that are relevant for human health. Therefore, the comprehensive assessment of long-term effects of reducing intake of energy (CALERIE-II) clinical trial was designed to test the effects of CR for 2 years on human physiology and potential predictors of health span and longevity (10). This study established that healthy adults in free-living conditions can only achieve 14% CR over a 2-year period (11). Importantly, CR in humans improved markers of cardiometabolic health and oxidative stress, including reduction in proinflammatory cytokines in blood without any measurable immunological tradeoffs (11–14). The CALERIE-II clinical trial established that simple reduction of calories, irrespective of meal composition, frequency, or timing of food intake lowered inflammation and enhanced metabolic health (15). Thus, the CALERIE study in healthy humans provided a platform to identify and harness biologically relevant potential CR mimetics that may serve as immunometabolic checkpoints of health span (15, 16).

The RNA-Seq analyses of adipose tissue from CALERIE-II participants led to the identification of a CR-regulated adipokine called secreted protein acidic and rich in cysteine (SPARC), also called osteonectin/BM-40 (17). SPARC is a secreted protein and a member of matricellular proteins that are modular, extracellular proteins binding to matrix proteins, receptors, and other cell surface–interacting molecules (18). SPARC is broadly expressed in multiple tissues, including bone and adipose tissues, and plays a role in tissue development and remodeling without being incorporated into insoluble ECM (19). Global deletion of SPARC during development revealed defects in bone formation, maintenance, and repair, as well as the role of SPARC in fibrosis (20, 21). Interestingly, obesity is associated with increased circulating SPARC levels in humans (22) and a high level of SPARC is associated with insulin resistance, inflammation, and diabetes (23, 24). In addition, *SPARC* variants are linked to alteration in fat distribution and glycemic control (25, 26). Moreover, elevated SPARC is positively associated with a proinflammatory M1-like macrophage phenotype (27). Interestingly, we recently reported that SPARC induces macrophage activation by inducing type-1 interferon-response in a toll-like receptor 4–dependent (TLR4-dependent) manner (17). However, how adipocyte-derived SPARC controls metabolism and inflammation during obesity and weight loss is unknown. Here, based on unbiased identification of SPARC as a CR-inhibited gene in human adipose tissue (17), we sought to determine whether adipocyte production of SPARC is a potential partial CR-mimetic target that can be harnessed to regulate metabolic inflammation during obesity.

## Results

### SPARC is a CR-inhibited adipokine.

**Figure 1:**
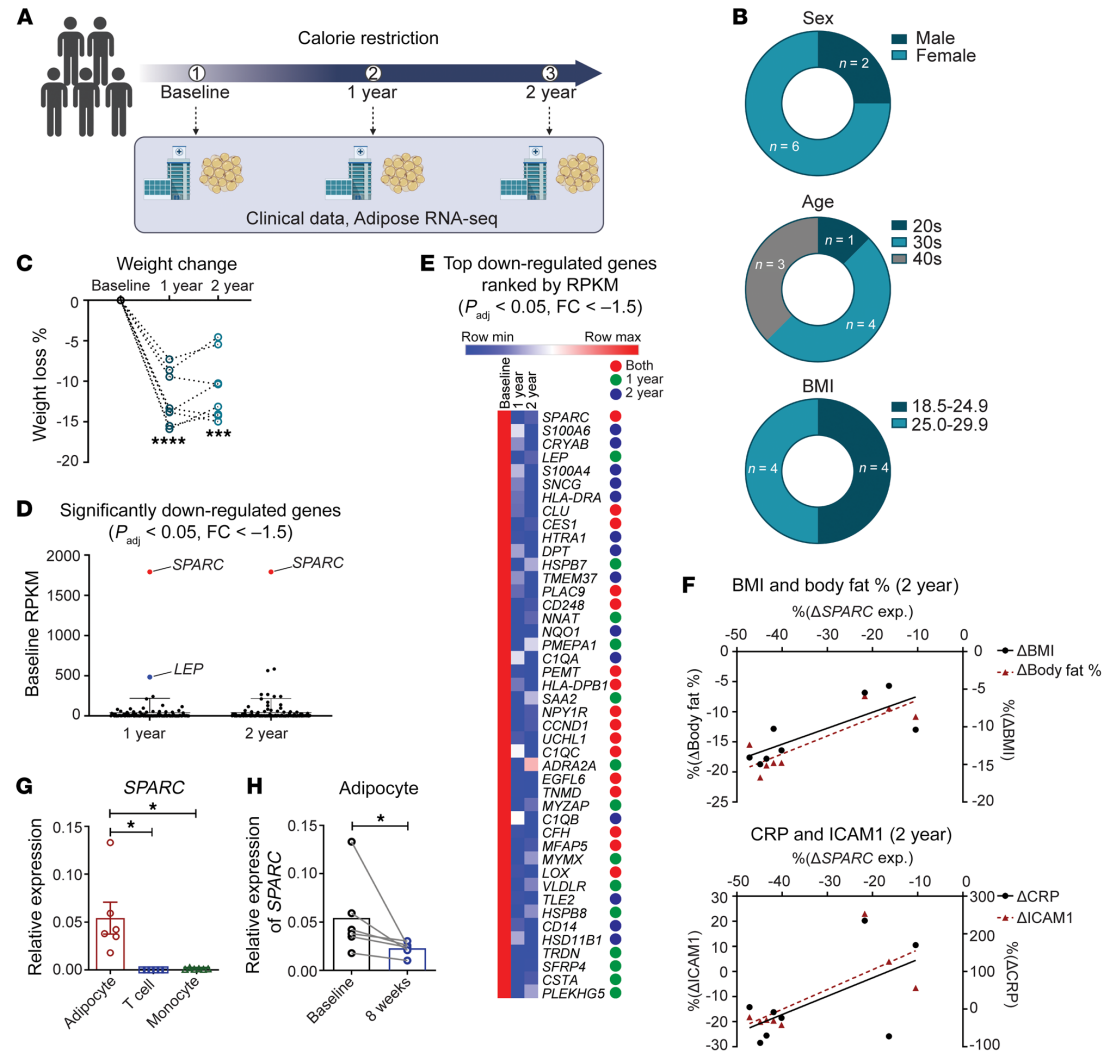
Inhibition of SPARC by CR is associated with improved metabolic outcomes in humans. (**A**) Study design and adipose tissue sample collection from human CALERIE-II study for the transcriptomic analyses. (**B** and **C**) Information of study participants that underwent 14% sustained CR and weight loss and provided adipose tissues for RNA-Seq. (**D**) Baseline RPKM of significantly downregulated genes (*P*_adj_ < 0.05, FC < –1.5) in RNA-Seq with human adipose tissue after 1 and 2 years of CR. (**E**) Heatmap of gene expression changes ranked by RPKM for top 30 genes that are significantly downregulated (*P*_adj_ < 0.05, FC < –1.5) from baseline to year 1 or year 2. The colored circles indicate genes that are significantly downregulated in 1 year only (green), 2 years (blue), or both (red). (**F**) Regression analyses between percentage changes of *SPARC,* normalized expression, and percent changes in BMI, body fat percentage (upper), CRP, and ICAM1 (lower) of participants with 2 years of CR (*n* = 8). (**G**) q-PCR analysis of *SPARC* in human adipocytes, T cells, and monocytes (*n* = 6). (**H**) q-PCR analysis of *SPARC* mRNA from primary adipocytes isolated from SAT of overweight children before and after 8 weeks of CR (*n* = 6). Error bars represent the mean ± SEM. 2-tailed unpaired and paired *t* tests (**C** and **H**), 1-way ANOVA with Dunnett’s multiple comparisons test for adjusted *P* values (**G**), and Pearson correlation analysis (**F**) were performed for statistical analysis. *P < 0.05; ***P < 0.001; ****P < 0.0001.

To discover adipocyte-derived factors that mediate the salutary effects of CR, we performed RNA-Seq of subcutaneous adipose tissue (SAT) from healthy human participants of the CALERIE-II study who underwent an average 14% sustained CR (Figure 1, A and B and Supplemental Table 1; supplemental material available online with this article; https://doi.org/10.1172/JCI169173DS1) (15). The weight loss after CR in all of the subjects that consented for adipose tissue biopsy collection revealed that between year 1 and 2, participants were weight stable (Figure 1C). Notably, the RNA-Seq revealed that *SPARC* expression is more than 3-fold higher than adipokine leptin in human adipose tissue (Figure 1D). In an effort to identify potential CR-mimetic targets, we focused on the most expressed and significantly downregulated genes with over 1.5-fold change after 1 or 2 years of CR. This bioinformatic analysis revealed that *SPARC* is the highest expressed gene at baseline among significantly downregulated genes after CR (Figure 1E). Among the most abundantly expressed genes in human adipose tissue of healthy humans with normal BMI, the top significantly downregulated genes at 1 and 2 years of CR are shown in Figure 1E. Interestingly, among the top genes, 3 genes inhibited by CR, (*SPARC*, *CRYAB,* and *CLU*) have previously been associated with improved metabolic outcomes after weight loss in individuals with obesity (28–30). These findings suggest that the CR-induced gene regulation patterns observed in adipose tissue of CALERIE-II participants may constitute core signature of negative energy balance, and, thus, could represent potential CR-mimetic targets (Figure 1E). Similar to the reduction of circulating leptin in study participants (31), CR significantly inhibited leptin mRNA in human adipose tissue (Supplemental Figure 1A). The change in circulating leptin level was positively correlated with reduction in *SPARC* adipose expression after CR (Supplemental Figure 1B). Consistent with the hypothesis that lowering of SPARC signals improved metabolic health in humans, a correlation analysis found that CR-induced decrease in *SPARC* is associated with reduction in BMI, fat mass, and circulating proinflammatory markers CRP and ICAM1 (Figure 1F).

SPARC is expressed broadly in several cell types including osteoblasts and microglia (19). Importantly, compared with adipocytes, immune cells like T cells and monocytes have low *SPARC* expression (Figure 1G), which cannot be further induced by T cell receptor (TCR) or TLR4 activation in these cell types (Supplemental Figure 1, C and D). Given the effects of CR on reduction of SPARC in whole adipose tissue of adults, we next investigated whether adipocyte-derived SPARC is regulated by dietary restriction–induced weight loss in overweight children. Similar to adults, adipocytes isolated from SAT of overweight children that underwent dietary restriction–induced weight loss demonstrated significant inhibition of *SPARC* expression (Figure 1H).

### Dietary regulation of adipose SPARC.

**Figure 2:**
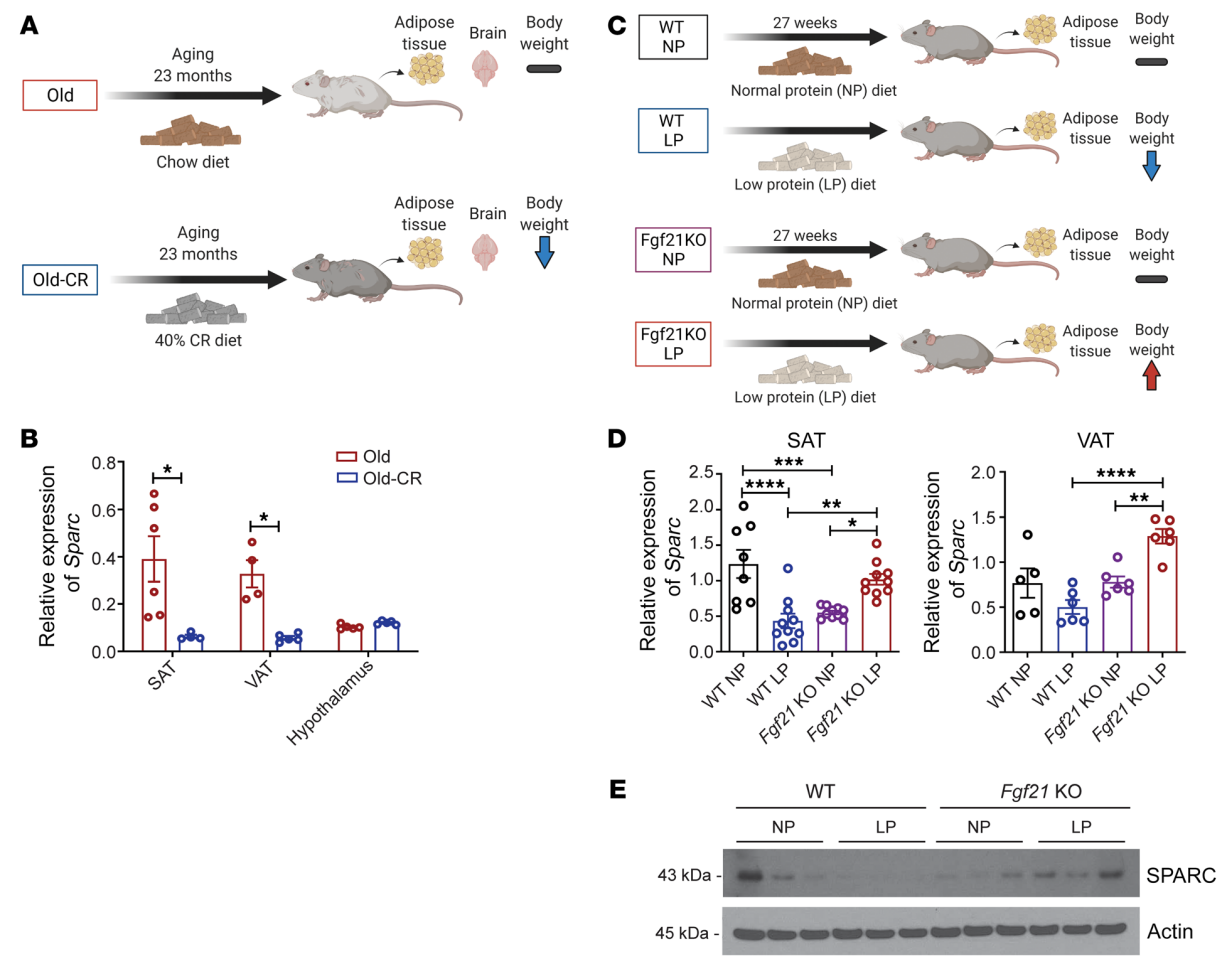
Dietary restriction reduces adipose SPARC levels in mice. (**A** and **B**) Schematic and the *Sparc* mRNA levels in subcutaneous adipose tissue (SAT) (*n* = 5, 4) and visceral adipose tissue (VAT) (*n* = 4, 5), and hypothalamus (*n* = 5, 5) in old mice (23 month) with or without life-long 40% CR. (**C**–**E**) Schematic and q-PCR analysis of *Sparc* mRNA (**C**, **D**), and protein immunoblot analysis (**E**) of WT mice on normal diet (WT NP), WT mice fed low-protein diet (WT LP) and mice lacking FGF21 fed normal (*Fgf21* KO NP) or low-protein diet (*Fgf2*1 KO LP). Error bars represent the mean ± SEM. 2-tailed unpaired *t* tests (**B**) and 1-way ANOVA test with Bonferroni’s multiple comparisons test for adjusted *P* values (**D**) were performed for statistical analysis. *P < 0.05; **P < 0.01; ***P < 0.001; ****P < 0.0001.

**Figure 3:**
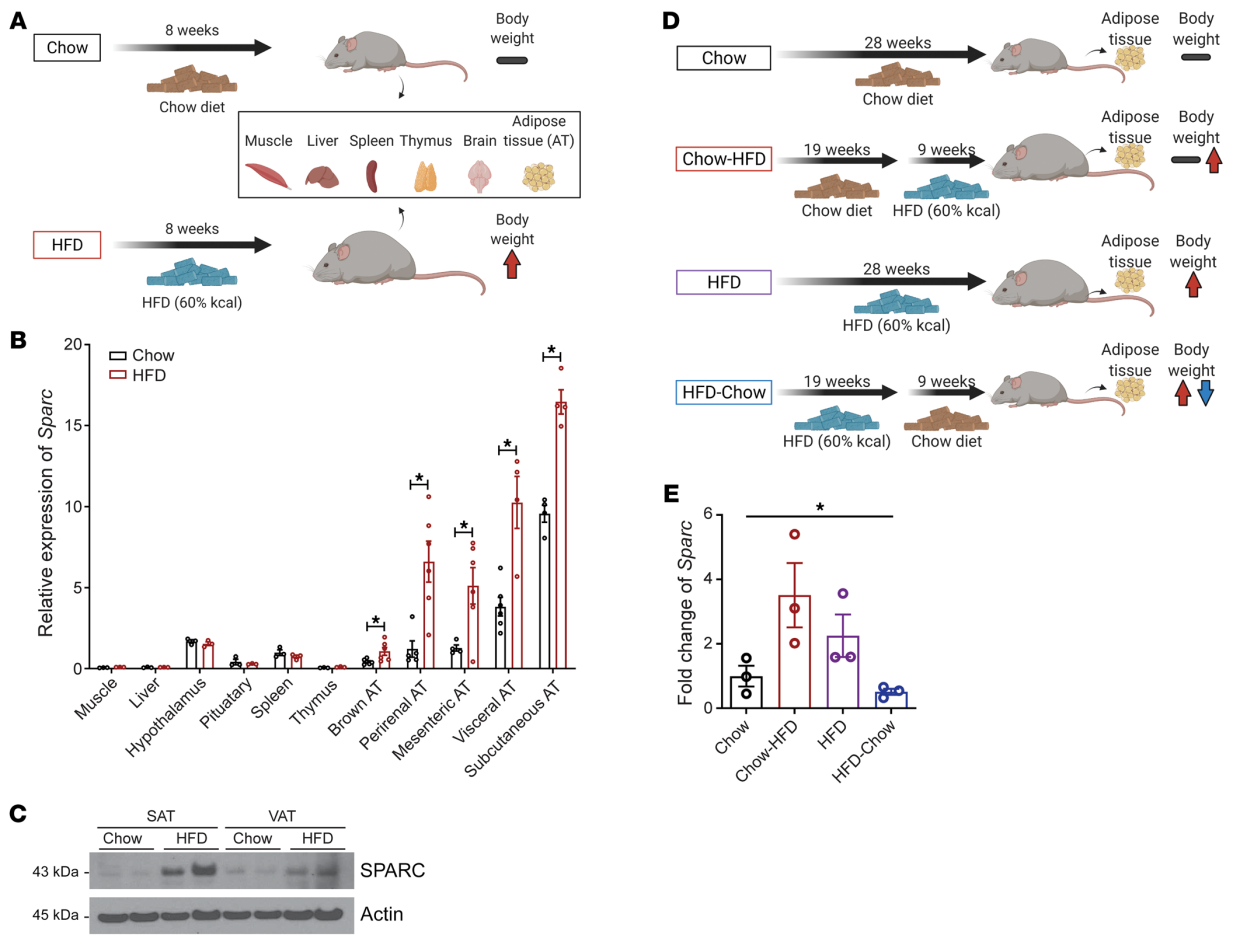
HFD increases adipose SPARC. (**A** and **B**) Schematic and Q-PCR analysis of *Sparc* mRNA in metabolic and immune tissues of mice fed chow (*n* = 3–6) and high-fat diet (HFD) (*n* = 3–6) for 8 weeks. (**C**) Immunoblot analysis of SPARC protein in SAT and VAT of mice fed chow and HFD for 8 weeks. (**D** and **E**) Schematic and q-PCR analysis of *Sparc* mRNA in mice fed HFD (60 Kcal% fat) and induced to undergo weight-loss by switching to control low calorie diet (14 Kcal% fat). Error bars represent the mean ± SEM. 2-tailed unpaired *t* tests (**B**) and 1-way ANOVA test with Tukey’s multiple comparisons test (**E**) were performed for statistical analysis. *P < 0.05.

To determine if SPARC is a bona fide partial CR mimetic, we next tested whether alterations in energy balance by diet regulates SPARC expression in mouse models, which may allow mechanistic investigation of its impact on metabolic health. Consistent with the findings from the CALERIE-II study, CR specifically decreased *Sparc* in subcutaneous and visceral adipose tissue (VAT) of 23-month-old mice (analogous to approximately 70-year-old humans) without affecting the expression in hypothalamus (Figure 2, A and B). Furthermore, similar to CR, low-protein (LP) diet, which is known to protect against adiposity and improve adipose tissue metabolism by increasing energy expenditure (EE) (32), caused significant reduction in *Sparc* mRNA (Figure 2, C and D) and protein expression (Figure 2E) in both subcutaneous and VATs. Importantly, fibroblast growth factor 21 (FGF21) is induced by LP diet and is required for coupling amino acid metabolism to improved metabolic outcomes (32). Interestingly, we found that, compared with control animals, LP diet–induced inhibition of *Sparc* is prevented in *Fgf21*-deficient animals, demonstrating an important role of FGF21 in regulation of SPARC in response to protein restriction (Figure 2E). Conversely, in high-fat diet–induced (HFD-induced) adiposity, *Sparc* mRNA and protein were specifically upregulated in adipose depots (Figure 3, A–C). Furthermore, weight loss induced by switching the mice from HFD to chow diet reversed the obesity-induced increase in *Sparc* (Figure 3, D and E). Together, these data establish that improvement of adipose tissue metabolism by dietary interventions such as LP feeding and CR are coupled with reduction of SPARC levels.

### Inducible inhibition of SPARC in adult mice reduces adiposity.

Early studies comparing WT nonlittermate controls to the *Sparc*-null animals on mixed C57BL6/129SVJ genetic background found that the developmental deletion of SPARC increased the size of adipose depots without affecting the total body weight (33). Recent studies using the *Sparc*-KO mice reported impaired glucose homeostasis in aging and HFD-induced obesity (34) with defects in insulin secretion from the pancreas (35). SPARC is also believed to be a myokine that is upregulated by exercise to inhibit colon tumorigenesis (36). Another in vitro study using cell lines reported a positive effect of SPARC on induction of lipolysis and thermogenesis (37). However, those reports contrast with many observations that increased SPARC levels are correlated with adiposity, metabolic dysregulation, and inflammation in human and rodent models (22, 24, 27, 38). Therefore, to test whether our findings of reduction of SPARC in adipose tissue in humans undergoing CR are causally linked to enhanced metabolic health, we developed new genetic mouse models to determine the role of SPARC in the context of obesity.

**Figure 4:**
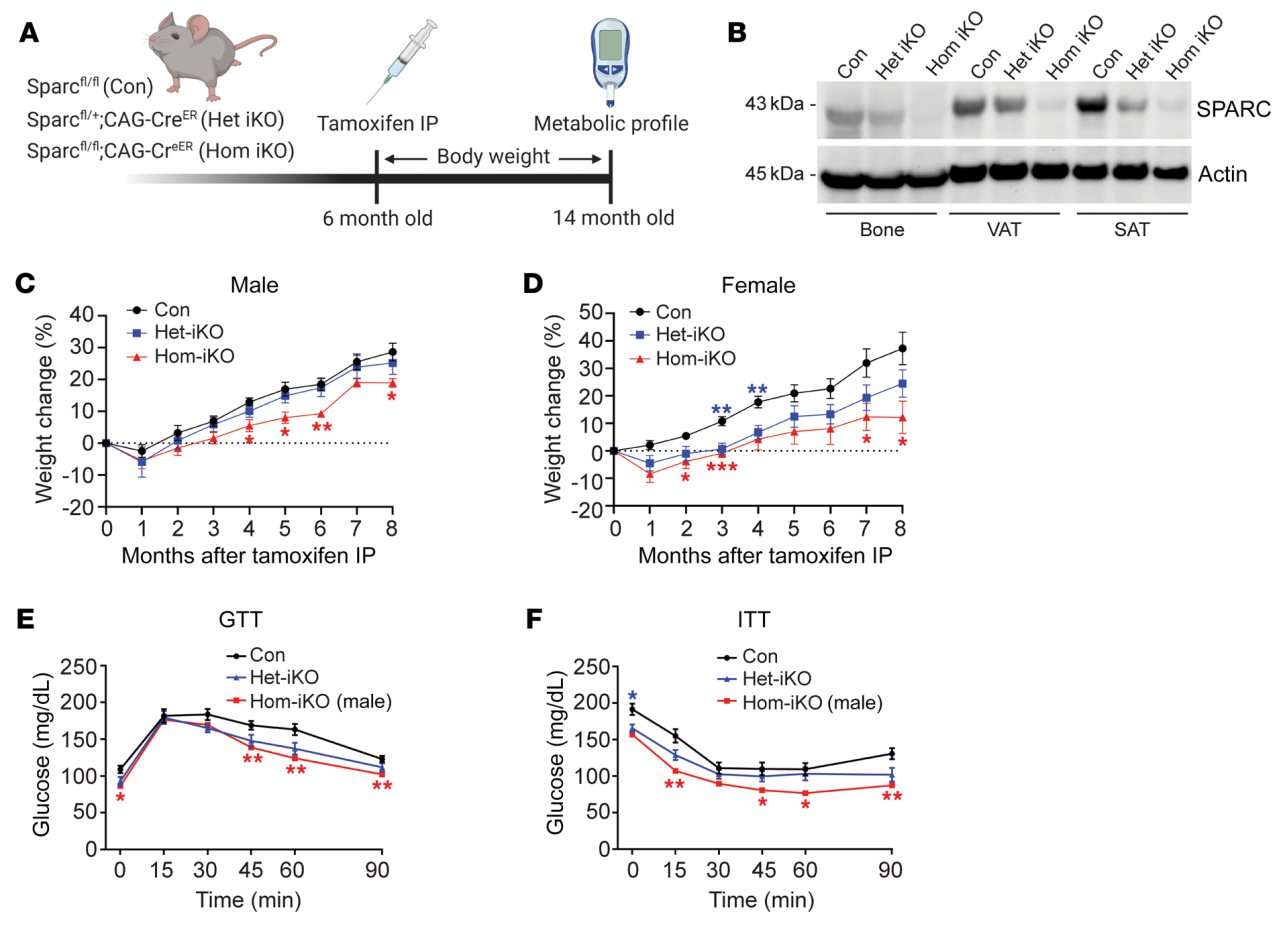
SPARC controls adiposity. (**A**) Schematic of experiments with inducible global *Sparc* KO mice. (**B**) Immunoblot analysis of SPARC protein in bone, VAT, and SAT in control *Sparc^fl/fl^* (Con), heterozygote (*Sparc*^fl/+^;CAG-Cre^ER^, Het iKO), and homozygote (*Sparc^fl/fl^*;CAG-Cre^ER^, Hom iKO) KO mice 6 weeks after tamoxifen injection. (**C** and **D**) Percentage of weight change of male (**C**) and female (**D**) littermate control (*n* = 10, 10), Het iKO (*n* = 10, 10), and Hom iKO (*n* = 5, 4) mice after tamoxifen injection. (**E** and **F**) Glucose tolerance test (GTT) (**E**), and insulin tolerance test (ITT) (**F**) of 14-month old (8 months after tamoxifen injection) male Con, Het iKO, and Hom iKO mice (*n* = 10, 10, 5). The blue star indicates statistical significance between Con and Het iKO mice, and the red star indicates statistical significance between Con and Hom iKO mice. Error bars represent the mean ± SEM. 2-way ANOVA test with Dunnett’s multiple comparisons test for adjusted *P* values (**C**–**F**) were performed for statistical analysis. *P < 0.05; **P < 0.01; ***P < 0.001.

**Figure 5:**
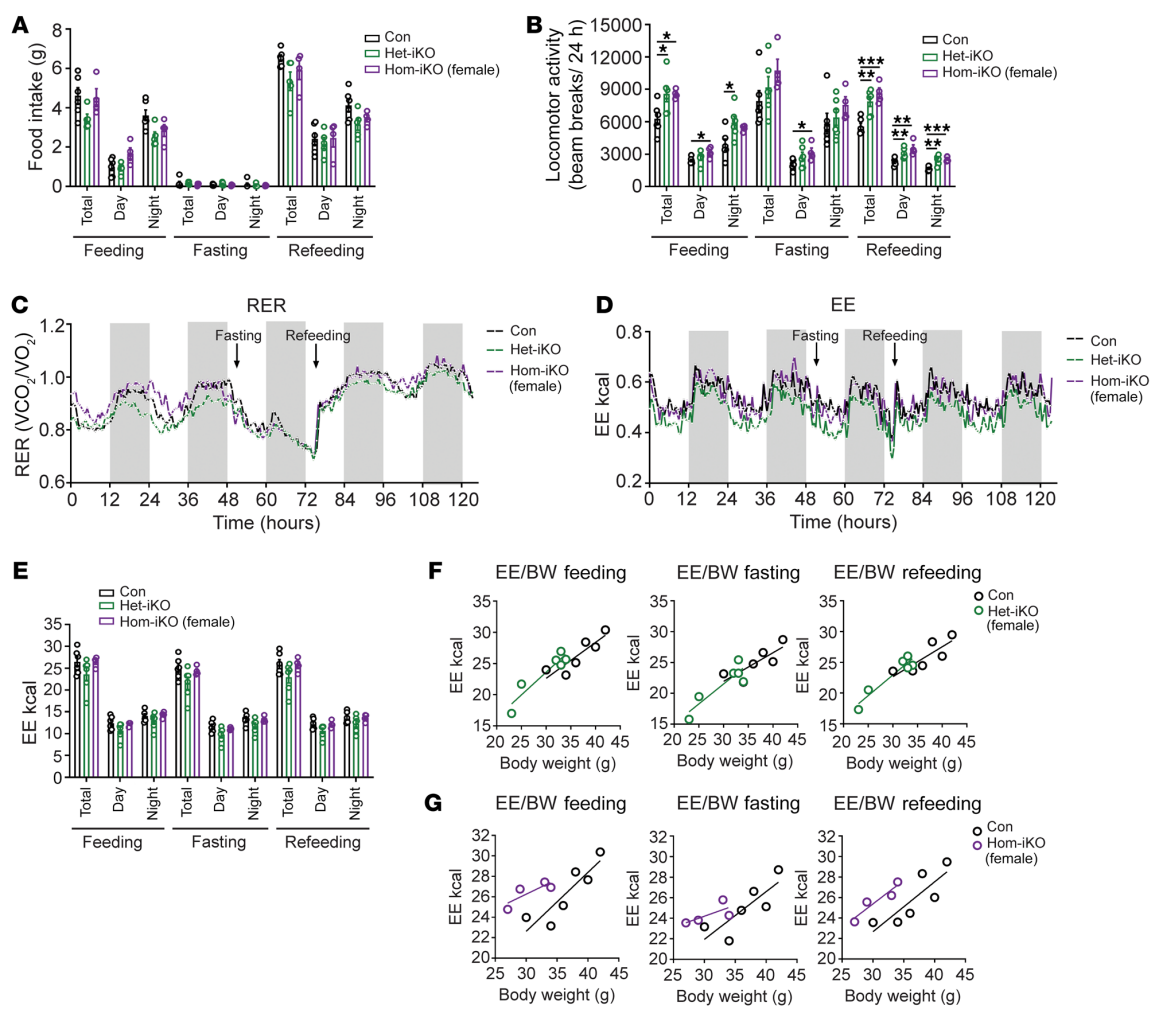
SPARC regulates EE in mice. (**A**–**C**) Metabolic cage analysis results of food intake (**A**), locomotive activity (**B**), and RER in 15-month old female Con, Het-iKO, and Hom-iKO mice (*n* = 6, 6, 4, respectively) (**C**). (**D** and **E**) Unnormalized EE by metabolic cage analysis of 15-month old female Con, Het-iKO, and Hom-iKO mice (*n* = 6, 6, 4, respectively). (**F** and **G**) Comparison of linear regression analyses about unnormalized energy expenditure (EE) and body mass between female Con and Het-iKO mice (*n* = 6, 6, respectively); ANCOVA (**F**) and between female Con and Hom-iKO mice (*n* = 6, 4, respectively) (**G**). Error bars represent the mean ± SEM. 2-tailed unpaired *t* tests were performed for statistical analysis (**A**, **B**, and **E**). *P < 0.05; **P < 0.01; ***P < 0.001.

To test the mechanism of SPARC in regulation of adipose tissue metabolism by avoiding genetic and developmental effects, we lowered SPARC expression in adulthood using tamoxifen-inducible CAG-Cre^ER^ (Supplemental Figure 2A). All studies were performed by comparing control littermates to heterozygote (Het-iSparc-KO) and homozygote KOs (Hom-iSparc-KO) on pure C57BL/6J background, where all groups were injected with tamoxifen to induce global reduction of SPARC in adult 6-month-old male and female mice (Figure 4, A and B). Interestingly, deletion of SPARC in adult life protected against increased body weight gain (Figure 4, C and D) until 8 months of age. These differences in body weight were mainly attributed to differences in fat mass (Supplemental Figure 2, B and C). Consistent with reduction in adiposity, inducible global deletion of SPARC in male and female mice improved glucose tolerance and insulin sensitivity (Figure 4, E and F). The reduction in adiposity after SPARC deletion was not due to a decrease in food intake during fasting or refeeding (Figure 5A and Supplemental Figure 2D), though Hom-i*Sparc*-KO mice displayed increased locomotion during the refeeding phase (Figure 5B and Supplemental Figure 2E).

It is known that in models of HFD-induced obesity in mice, energy intake is the primary driver of positive energy balance leading to adiposity (39). Indeed, consistent with Newton’s second law of motion — that higher EE is required to move greater body mass — the obese mice on HFD have increased EE and are in positive energy balance due to increased intake of high calorie diet (39). Given that SPARC deletion did not affect energy intake, we therefore calculated EE as a variable that could account for reduced adiposity upon inducible *Sparc* knockdown. As expected, when normalized to total body weight and compared with control animals, SPARC-deficient mice had higher EE (Supplemental Figure 2, L–O), without any change in respiratory exchange ratio (RER) (Figure 5C and Supplemental Figure 2P). However, division of EE by body weight overcompensates for the mass effect (40). Using ANCOVA with body weight as covariate suggested that deletion of SPARC caused modest increase in unnormalized EE when compared as linear regression analysis (40, 41) in female Hom-i*Sparc*-KO mice at baseline, during fasting, and refeeding conditions (Figure 5, D–G and Supplemental Figure 2, Q–T). Thus, increased locomotion together with modest increase in EE over an extended period could account for the net decrease in adiposity. Together, these data show that reduction of SPARC in adult life protected against adiposity and may have conferred partial CR-like metabolic benefits on adipose tissue metabolism.

### Adipokine SPARC regulates energy metabolism.

**Figure 6:**
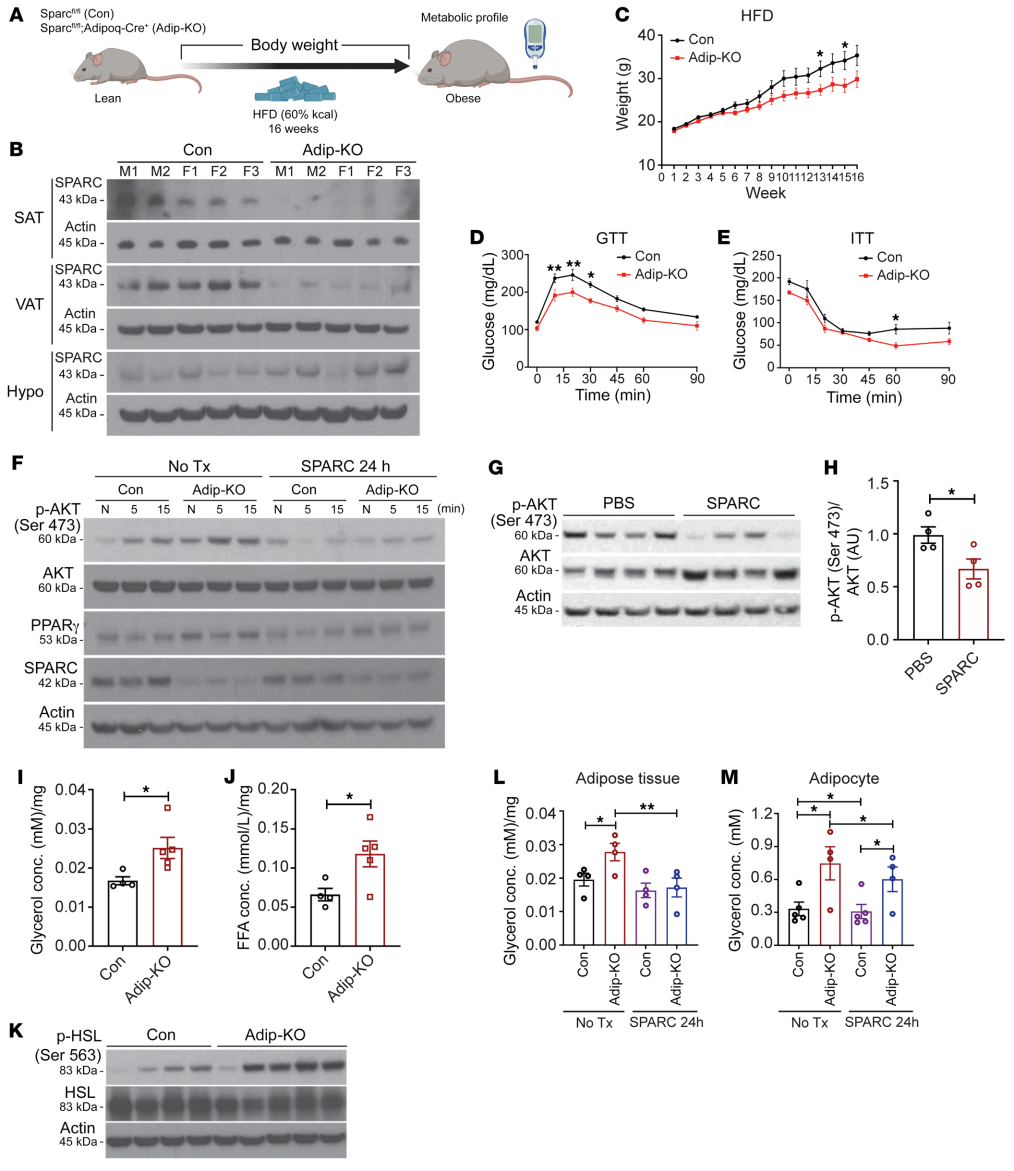
Reduction of adipocyte-derived SPARC improves metabolic health. (**A**) Schematic of experiments using *Sparc* floxed mouse (Con) and adipocyte specific *Sparc* KO mouse (Adip-KO). (**B**) Immunoblot analysis of SPARC protein in SAT, VAT, and hypothalamus (Hypo) in Con and Adip-KO mice. (**C**) Weight change in Con and Adip-KO female mice during 16 weeks of HFD (*n* = 12, 12, respectively). (**D** and **E**) GTT (**D**) and ITT (**E**) in Con and Adip-KO mice with 16 weeks of HFD (*n* = 7, 7, respectively). (**F**) Insulin (100 nM) response signaling at the indicated time in cultured primary adipocytes from Con and Adip-KO mice with or without SPARC (20 μg/mL) treatment for 24 hours. “N” indicates no treatment. (**G** and **H**) Immunoblot analysis and quantification of AKT phosphorylation (Ser 473) 5 minutes after insulin injection. The mice were pretreated with SPARC (100 μg) by i.p. injection 12 hours before the insulin injection and tissue collection. (**I** and **J**) Glycerol (**I**) and free fatty acid (FFA) (**J**) levels in ex vivo lipolysis assay with VAT from Con and Adip-KO mice after 24 hours of fasting (*n* = 4, 5, respectively). (**K**) Immunoblot analysis for lipolysis signaling in adipose tissue explants (SAT) from Con and Adip-KO mice after 24 hours of fasting (*n* = 4, 5, respectively). (**L**) Glycerol assay of SAT explants from Con and Adip-KO mice with or without ex vivo SPARC treatment (20 μg/mL) for 24 hours followed by stimulation with 10 μM norepinephrine (NE) (*n* = 4, 4, respectively). (**M**) Glycerol assay in differentiated adipocytes from adipose SVF of Con and Adip-KO mice with or without preincubation of SPARC (20 μg/mL) for 24 hours (*n* = 5, 4, respectively). The adipocytes were treated with NE (10 μM) for 4 hours to activate lipolysis and glycerol levels were measured in supernatants. Error bars represent the mean ± SEM. 2-tailed unpaired (**C**, **H**, **I**, and **J**), paired (**L** and **M**) *t* tests, and 2-way ANOVA test with Bonferroni’s multiple comparisons test for adjusted *P* values (**D** and **E**) were performed for statistical analysis. *P < 0.05; **P < 0.01.

SPARC is highly expressed in multiple cell types such as osteoblasts, fibroblasts, and adipocytes in the periphery and microglia in the brain (42, 43). Given that the inducible whole-body knockdown of SPARC mimics some of CR’s beneficial effects on adiposity, we next investigated whether reduction of SPARC in adipocytes (Figure 6A) was sufficient to drive the immunometabolic effects of this matricellular protein. Adiponectin-Cre efficiently and specifically deleted SPARC from adipose depots without affecting the expression in the hypothalamus (Figure 6B). Deficiency of SPARC in adipocytes did not impact the body weight or adiposity in mice fed control chow diet (Supplemental Figure 3, A and B). Interestingly, compared with littermate controls, adipocyte-specific *Sparc*-KO mice (Adip-KO) were protected from HFD-induced obesity (Figure 6C) and displayed improved glucose tolerance (Figure 6D) and insulin-sensitivity (Figure 6E). SPARC’s reduction in adipocytes and subsequent response on adiposity in mice were specific to females with HFD, as male animals displayed similar body weight and insulin sensitivity in response to HFD feeding (Supplemental Figure 3, B–I). This is consistent with more pronounced difference in female Hom-i*Sparc*-KO mice with inducible global reduction of SPARC. These data suggest a sexually dimorphic response of adipocyte-derived SPARC upon organismal metabolism in C57BL/B6J mice.

To investigate whether increased levels of SPARC directly affect insulin signaling independently of weight loss, we isolated preadipocytes and differentiated them into mature adipocytes from control and adipocyte-specific *Sparc*-KO mice and exposed them to insulin together with recombinant SPARC protein. Compared with *Sparc^fl/fl^* (Con) adipocytes, SPARC-deficient adipocytes (Adip-KO) displayed increased AKT activation and PPARγ expression suggesting improved insulin signaling (Figure 6F). Conversely, recombinant SPARC treatment of adipocytes reduced AKT phosphorylation (Figure 6F), which is consistent with a prior report that SPARC overexpression induced insulin-resistance in the 3T3L1 adipocyte cell line (38). In addition, in vivo gain of function by exogenous SPARC injection into WT mice demonstrated reduced AKT activation in adipose tissue (Figure 6, G and H). Collectively, these results show that downregulation of SPARC promoted insulin sensitivity in adipocytes.

### SPARC controls adipocyte lipolysis.

To determine how lowering SPARC protects against diet-induced obesity, we next investigated changes in energy balance upon downregulation of SPARC in adipocytes using indirect calorimetry. These experiments revealed that, compared with control littermates on a HFD, the adipocyte-specific *Sparc*-KO mice showed moderately increased unnormalized EE on HFD, especially at some time points at night (Supplemental Figure 4A). The ANCOVA analysis with body weight as covariate showed no change in EE during the day and modest elevation of EE in *Sparc* adipocyte–KO mice at night (Supplemental Figure 4B). As expected, this effect of increased EE in *Sparc* adipocyte–KO mice was significantly higher when body weight was normalized to EE (Supplemental Figure 4, C–F). Like global inducible–KO mice, there was no significant difference in food intake (Supplemental Figure 4G) between *Sparc^fl/fl^* and *Sparc* adipocyte–KO mice with HFD. Given that energy intake, water consumption, and locomotive activity (Supplemental Figure 4, G–I) in *Sparc* adipocyte–KO mice were not significantly different from *Sparc^fl/fl^* mice, a modest increase in night time EE may partially account for reduced adiposity as we observed in global inducible–KO mice. Male mice lacking SPARC in adipocytes displayed no significant change in EE, food intake, or locomotive activity (Supplemental Figure 4, J–L).

Reduced adiposity as well as increased EE requires hydrolysis of adipocyte triglycerides into fatty acids through the process of lipolysis (44). To investigate the effect of SPARC deficiency on lipolytic activity of adipocytes, we fasted *Sparc^fl/fl^* and *Sparc* adipocyte–KO mice for 24 hours and measured the lipolytic activity of adipose tissue. Consistent with reduced adiposity, the adipocyte-specific SPARC–deficient mice had higher lipolytic response compared with *Sparc^fl/fl^* mice as measured by increased release of glycerol, free fatty acid (FFA), and activation of hormone-sensitive lipase (p-HSL Ser564) (Figure 6, I–K and Supplemental Figure 4, M–O). In support of these findings, we next took an ex vivo approach, whereby adipose tissue explant was primed with norepinephrine and treated with recombinant SPARC protein, which demonstrated a significant reduction of glycerol release (Figure 6L). To further investigate whether SPARC acts directly on adipocytes to regulate lipolysis, in vitro differentiated adipocytes from *Sparc^fl/fl^* and adipocyte-specific *Sparc*–KO mice were treated with a β-adrenergic agonist together with exogenous recombinant SPARC (Figure 6M). Consistent with results from the adipose tissue explant, the catecholamine-induced glycerol release from adipocytes was reduced by SPARC treatment (Figure 6, L and M). Collectively, these data demonstrate that reduction in SPARC production from adipocytes protected against obesity by decreasing adiposity with modest increases in EE and enhanced lipolytic response in female mice.

### Reduction of adipocyte-derived SPARC induces weight loss.

**Figure 7:**
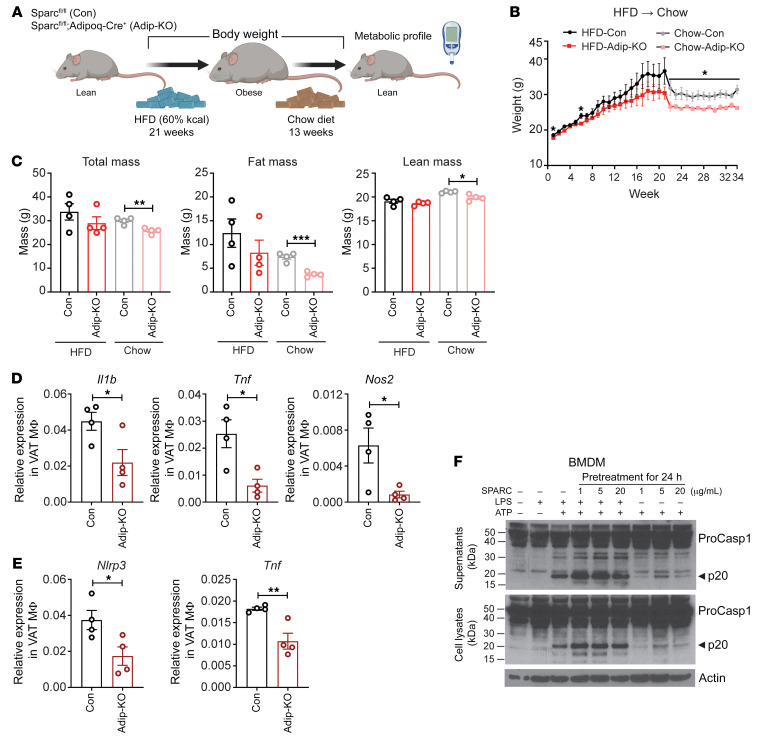
Adipocyte-derived SPARC controls macrophage inflammation. (**A** and **B**) Schematic and weight change of Con and Adip-KO mice with 21 weeks of HFD followed by 13 weeks of chow diet (*n* = 5, 5, respectively). (**C**) Body composition analysis of female Con and Adip-KO mice before and after diet change from HFD to chow diet (*n* = 4, 4, respectively). (**D** and **E**) q-PCR analysis of inflammatory gene (**D**) and components of inflammasome (**E**) in VAT macrophages (F4/80^+^) in obese mice switched to chow diet (*n* = 4, 4, respectively). (**F**) Inflammasome activation after pretreatment of SPARC protein for 24 hours following ATP (5mM) treatment with or without LPS (1 μg/mL) measured by caspase-1 Western blot analysis in cell lysate (lower) and supernatant (upper). Error bars represent the mean ± SEM. 2-tailed unpaired *t* tests were performed for statistical analysis. *P < 0.05; **P < 0.01; ***P < 0.001.

Given that sustained CR-induced weight loss in humans is coupled with reduction in adipose SPARC expression, we next investigated if adipocyte-derived SPARC regulates adiposity and weight loss in mice (Figure 7A). The control littermates (Con) and adipocyte SPARC-deficient animals (Adip-KO) were fed HFD for 21 weeks and then switched to chow diet for additional 13 weeks to induce weight loss. Consistent with the above results, adipocyte-specific *Sparc*–KO mice were protected from obesity and maintained significantly lower weight than WT controls during weight loss (Figure 7B) with significant reduction of fat mass (Figure 7C). Consistent with HFD, the difference in weight loss after the diet switch was specific to female adipocyte-specific *Sparc*–KO mice and not observed in male animals (Supplemental Figure 5, A and B).

Considering the increase of SPARC during HFD and the known role of NLRP3 inflammasome-driven inflammation in causing metabolic dysfunction (45), we tested whether SPARC could contribute to macrophage-derived inflammation. Interestingly, reduction of SPARC level in adipose tissue with diet change–induced weight loss reduced the inflammatory cytokines as well as inflammasome components in adipose tissue macrophages (ATMs) (Figure 7, D and E), suggesting that reduction in SPARC lowered adipose inflammation. Taken together, SPARC contributes to adipose health through adipocyte-autonomous mechanisms by affecting insulin sensitivity, as well as indirectly through decreased adipose inflammation.

### SPARC primes and activates the inflammasome in macrophages.

Given that obesity-related increase in NLRP3 inflammasome activation is a predominant regulator of IL-1β production and decreased health span (45-47), we next tested whether SPARC gain of function impacts the inflammasome. Exposure of bone marrow–derived macrophages (BMDM) with recombinant SPARC failed to enhance or activate caspase-1 with or without cotreatment of LPS or ATP (Supplemental Figure 5C). However, in TLR4-primed BMDMs pretreated with SPARC and extracellular ATP caused elevation of inflammasome activation (Figure 7F). Interestingly, SPARC serves as a priming signal for the NLRP3 inflammasome in the absence of LPS because treatment of BMDM with SPARC and ATP caused increased caspase-1 cleavage, but to a lesser degree than LPS (Figure 7F). These data are consistent with our recent finding that SPARC activates TLR4 (17), which is a critical step for inflammasome priming.

**Figure 8:**
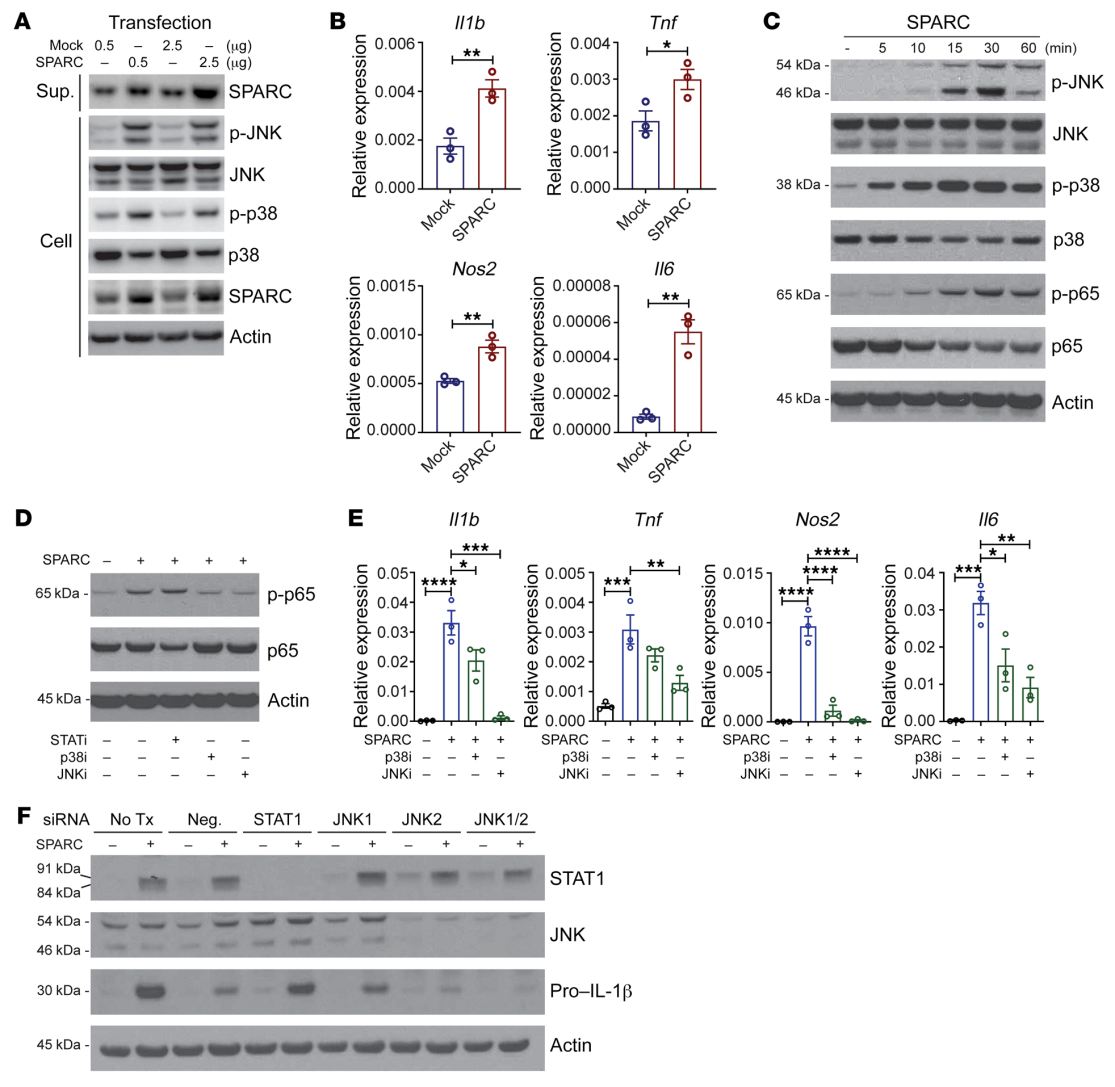
SPARC activates inflammation in macrophages via JNK signaling. (**A**) Human *SPARC* or mock vector was overexpressed in RAW 264.7 cells by transient transfection (0.5 and 2.5 μg) and Western-blot analyses of SPARC, JNK and p38 MAPK are shown. The experiment was repeated in triplicate and performed twice. (**B**) q-PCR analysis of *Il1b*, *Tnf*, *Nos2*, and *Il6* in RAW 264.7 cells with *SPARC* or mock vector overexpression. (**C**) Primary BMDMs were treated with SPARC (5–60 minutes) and JNK, p65 NF-κB, and p38 MPAK were quantified by immunoblot analysis. (**D**) Representative immunoblot of p-p65 NF-κB in BMDMs pretreated with STAT1, p38, and JNK inhibitor and in presence of SPARC (20 μg/mL). (**E**) q-PCR analysis of *Il1b*, *Tnf*, *Nos2*, and *Il6* in BMDMs pretreated with p38 or JNK inhibitor followed by SPARC treatment (20 μg/mL). (**F**) Primary BMDMs were transfected with JNK and STAT1 siRNA and immunoblot analysis was performed to quantify Pro-IL-1β protein levels. The experiment was repeated in triplicate and performed twice. Error bars represent the mean ± SEM. 2-tailed unpaired *t* tests (**B**) and 1-way ANOVA test with Bonferroni’s multiple comparisons test for adjusted *P* values (**E**) were performed for statistical analysis. *P < 0.05; **P < 0.01; ***P < 0.001; ****P < 0.0001.

To gain further insights into the mechanism of action of SPARC in control of macrophage inflammation, we next investigated the downstream signaling machinery activated by SPARC in macrophages. The recombinant SPARC used in our study was derived from Chinese hamster ovary (CHO) cells that allow proper protein folding without any LPS contamination. As an additional specificity control, we next genetically overexpressed *SPARC* in myeloid cell lines. Compared with mock transfected cells, the transient overexpression of *SPARC* in mouse RAW 264.7 myeloid cell line successfully increased the SPARC protein expression within cells and cell culture supernatants (Figure 8A). Interestingly, gain of SPARC function in RAW 264.7 cells caused increased phosphorylation of JNK and p38 MAPK and higher expression of proinflammatory mediators, *Il1b*, *Tnf*, *Nos2*, and *Il6* (Figure 8, A and B). Consistent with these data, treatment of BMDMs with recombinant SPARC caused a time-dependent increase in phosphorylation of p65 NF-κB, p38 MAPK, and JNK (Figure 8C). Interestingly, SPARC-induced phosphorylation of p65 NF-κB was reduced in the presence of p38 MAPK and JNK inhibitors and was STAT1 independent (Figure 8D). Consistently, the expression of proinflammatory genes was reduced by pretreatment of p38 MAPK and JNK inhibitors (Figure 8E). Furthermore, RNAi-mediated knockdown of JNK1 and 2 in primary macrophages prevented SPARC-induced upregulation of IL-1β (Figure 8F). Indeed, JNK activation in macrophages is an important driver of metabolic inflammation and impaired insulin signaling in obesity (48). Our data also demonstrate that reduction of adipocyte-derived SPARC acted on ATMs in fat to decrease obesity-related inflammation.

## Discussion

Increased levels of SPARC are associated with metabolic diseases — including obesity, diabetes, and cardiovascular disease (22, 23) — that are associated with chronic inflammation. Based on our prior findings that CR reduced SPARC expression in human adipose tissue, we hypothesized that adipocyte-derived SPARC controls energy metabolism and acts on macrophages to inhibit inflammation. Given SPARC’s established role as a matricellular protein, we reasoned that reduction of SPARC by CR controls adipose tissue remodeling and calibrates macrophage activation. The physiological rationale of release of proinflammatory cytokines by tissue resident macrophages during infection is well established, but the origin of inflammation in obesity and aging are still incompletely understood. We and others have shown that the NLRP3 inflammasome is a major mechanism that controls macrophage-derived inflammation in obesity (45, 46). Consistent with recent findings that SPARC activates TLR4 (17), we found that SPARC induced NLRP3 inflammasome-mediated inflammation by serving as a priming signal involving JNK-p38 and p65 activation. In line with our studies, similar to the NLRP3-KO mice, the SPARC-deficient mice are also protected from cardiovascular disease with lower inflammation and reduced macrophage infiltration (27).

SPARC has been studied in cancer and bone biology for a long time; indeed, SPARC affects the prognosis of some types of cancers and bone tissue development and maintenance, as determined by discovery of mutations and mouse-model studies. Considering that there is shared fundamental biology between SPARC’s role on adipose tissues during obesity and its role on cancer and bone microenvironments, it is possible that SPARC may affect cancer progression or bone remodeling by acting on resident myeloid cells. The cell-cell interaction effects by SPARC in specific tissues can be further investigated in future studies using appropriate model systems.

Intriguingly, the effect of adipocyte-derived SPARC in mice were specific to females and not males. Most studies are performed on male mice, and sexually dimorphic immunometabolic responses in females are understudied and often ignored. We identified HFD-induced metabolic dysregulation in both male and female mice in this study and female-specific protective effects by reduction of adipocyte-derived SPARC were identified. Future studies are required to determine the mechanisms underlying the biology of sexually dimorphic effects on C57BL/B6J mice. Importantly, in CALERIE-II, both male and female study participants showed significant reduction in adipose SPARC, which was associated with improved metabolic outcomes. So far, the data from human studies do not show sex-specific effects of reduction of SPARC and outcomes on weight loss or weight gain. It is possible that the sexually dimorphic response of SPARC on immunometabolism observed in rodents could likely reflect differences specific to mouse strains. These data further underscore that our reverse translation approach and unbiased identification of drivers of CR mechanism from randomized controlled human clinical trials like CALERIE can reveal CR-mimetic immunometabolic checkpoint targets that are relevant to human biology.

Our study highlights that reduction of adipocyte-derived SPARC dampens inflammation that may serve as potential partial CR-mimetic. Consistent with our data, the reduction of SPARC in *Drosophila* prolongs lifespan, while complete knockout of SPARC was embryonic lethal (49). In accordance with our results, reduction of SPARC production from adipocytes during aging in mice who were fed normal chow diet also protects against inflammation and extended health span (17). In summary, our study provides proof-of-concept evidence that CR in human highlights immunometabolic checkpoints that can be harnessed to target obesity and metabolic inflammation. We identified SPARC, a CR-inhibited matricellular adipokine that serves as one of the CR-mechanisms to reduce inflammation and improve metabolic health. Given that SPARC is a secreted protein, future studies to neutralize this matrikine through specific monoclonal antibodies may offer potential approaches to target NLRP3-driven inflammation and obesity-associated diseases.

## Methods

### Human samples.

The participants in this study were part of the CALERIE phase II (10) study, which was a multi-center, parallel-group, randomized controlled trial by recruitment of individuals who were nonobese and healthy. Over two hundred individuals from Pennington Biomedical Research Center, Washington University (St. Louis, Missouri, USA) and Tufts University (Boston, Massachusetts, USA) participated in the study (NCT00427193). Duke University (Durham, North Carolina, USA) served as a coordinating center. This study was planned to investigate the effects of 2 years of CR on health, including aging-related immune and metabolic phenotypes. The intervention was designed to achieve and maintain a weight loss of 25% through decreased caloric intake even though they actually reached 14% of CR (11). After baseline assessments, participants were randomly assigned to 25% calorie restriction (CR group) or ad libitum calorie intake (Control group) for 2 years. Men were between 20 and 50 years old and women were between 20 and 47 years old. Their BMIs were between 22.0 and 27.9 kg/m^2^ at the initial visit. Samples were collected at baseline, after 1 year, and after 2 years of intervention. Abdominal SAT biopsy was performed on a portion of CR group participants and used for RNA-Seq in this study.

### Human cells.

For human adipocyte samples with diet restriction, participants were provided with 1,200 calories for 8 weeks to promote weight loss. This study was performed with an approval by the ethics committees of the Yale University Hospital. The protocol number is 1007007067 and the clinical trial number is NCT01901978. Following SAT biopsy and collagenase digestion, the adipocytes in the floating cell fraction were collected and isolated by depletion of contaminating cells including immune cell types using a mixture of biotinylated antibodies against CD45 (1:400; 304004, Biolegend), CD34 (1:200; 13-0349-82, eBioscience), and CD31 (1:5; 13-0319-82, eBioscience). The adipocytes were used for RNA isolation and real-time reverse-transcription PCR. Human T cell and monocytes were isolated from PBMC and activated with CD3/CD28 antibodies (11452D; Thermo Fisher Scientific) and LPS (1 μg/mL for 24 hour; Sigma-Aldrich) respectively.

### Animal care.

All mice used in this study were housed in specific pathogen-free facilities in ventilated cage racks that deliver HEPA-filtered air to each cage with free access to sterile water through a Hydropac system at Yale School of Medicine. Sentinel mice in our animal rooms were negative for currently tested standard mouse pathogens (Ectromelia, EDIM, LCMV, Mycoplasma pulmonis, MHV, MNV, MPV, MVM, PVM, REO3, TMEV, and Sendai virus) while the studies were performed (Pathogen tests by Research Animal Diagnostic Laboratory). Mice were fed a standard vivarium chow (Harlan, 2018s) unless special diet was provided, and housed under 12-hour light/dark cycles.

### Mouse models.

C57BL/6J (WT) mice were bred from our lab colony or purchased from Jackson Laboratories. *Sparc* adipocyte–KO mice were generated and bred in our lab. ES cells of *Sparc*^tm1a(EUCOMM)Wtsi^ transgenic mice with C57BL/6 background were purchased from European Mouse Mutant Cell Repository (EuMMCR) and injected at Yale Genome Editing Center to make a transgenic construct harboring heterozygote mice. The mice were bred with flippase transgenic mice to remove reporters and selection markers of the transgenic construct, followed by crossing with Adiponectin-Cre^+^ mice to generate adipocyte-specific *Sparc*–KO mice. Littermate control mice (*Sparc^fl/fl^*) were used for WT controls in all experiments.

For the HFD experiment, mice were fed with HFD (Research Diets, D12492i, 60% kcal as fat). The HFD started at 6 weeks of age and was maintained up to 16 weeks. The weight of the mice was measured every week.

For the experiment with the diet change condition, the mice were fed with chow or HFD at 6 weeks of age and maintained for 19 or 21 weeks. The diet was either changed to HFD from chow or to chow from HFD for the following 9 or 13 weeks. The weight of the mice was measured every week.

For old mice in the CR condition, mice were obtained from National Institute on Aging (NIA). The mice were 23-months old and were fed with either chow or CR diet (NIH31-Fortified) at NIA. The CR diet was gradually provided from 10% restriction at 14 weeks, 25% restriction at 15 weeks, and to 40% restriction at 16 weeks. CR was maintained until the mice were sacrificed.

For mice with the protein-restriction diet, the experiment was performed as described previously (32). FGF21-deficient mice on the C57BL/6J background were provided by Dr. Steven Kliewer (UT Southwestern; Dallas, Texas, USA). Control and LP diets (Research Diets) were made to be isocaloric by changing the amount of protein and carbohydrate while keeping the fat content constant. For protein source, the control diet contained 20% casein by weight, whereas the LP diet contained 5% casein. Regarding energy basis, the control diet contained protein at 18%, while the LP diet contained 4%.

### Adipose tissue processing and stromal vascular fraction collection.

After the mice were euthanized, visceral and subcutaneous adipose were collected. Each adipose tissue was enzymatically digested in 0.1% collagenase I or II (Worthington Biochemicals) in HBSS (Life Technologies) for 45 minutes at 37°C. The stromal vascular fraction (SVF) was collected by centrifugation at 525*g* for 10 minutes, then washed and filtered by 100 μm and 70 μm strainers. RBCs were lysed using ACK lysis buffer (Quality Biological). Cells in SVF were resuspended in 1 mL of RPMI (Thermo Fischer Scientific) with 10% FBS (Omega Scientific) and 1% antibiotics/antimycotic (Thermo Fischer Scientific) for counting before further experiments.

### Adipocyte culture.

The pellet of SVF from SAT including preadipocytes was resuspended in DMEM/F-12 Glutamax, (Thermo Fischer Scientific) with 10% FBS (Omega Scientific) and 1% antibiotics/antimycotic (Thermo Fischer Scientific) and plated in 6-well plates. Media was replaced every other day until cells were confluent. The cells were incubated with media containing 0.5 mM isobutylmethylxanthine (Sigma-Aldrich), 125 nM indomethacin (Sigma-Aldrich), 5 μM dexamethasone (Sigma-Aldrich), 850 nM insulin (Sigma-Aldrich), 1 nM T3 (Sigma-Aldrich), and 1 μM rosiglitazone (Cayman Chemical) for 2 days to induce differentiation. Then, the cells were incubated with maintaining media containing 850 nM insulin, 1 nM T3 and 1 μM rosiglitazone until to be used for experiments (Day 13–17). Maintaining media was replaced every other day. For the experiment to measure activity of insulin signaling, 20 μg/mL of SPARC recombinant protein (PeproTech) was preincubated for 24 hours before 2 hours of starving followed by 100 mM insulin (Sigma-Aldrich) treatment for 5 minutes or 15 minutes.

### Cell-line culture and transfection.

The RAW 264.7 cell line was purchased from ATCC (ATCC TIB-71). The cells were cultured in DMEM with high glucose (Thermo Fischer Scientific) including 10% FBS (Omega Scientific) and 1% antibiotics/antimycotic (Thermo Fischer Scientific) and plated in 24-well plates as 1 × 10^6^ cells per well. The plated cells were transfected with a vector containing human *SPARC* ORF or a mock vector (GeneCopoeia) using Lipofectamine 3000 (Thermo Fisher Scientific) following manufacturer’s instruction. After 4 hours of transfection, the media was replaced and cells were collected with RIPA buffer or RLT buffer (Qiagen) for Western blot or quantitative-PCR (qPCR) analysis after 48 hours of transfection.

### Macrophage isolation.

For positive selection of macrophages, SVF from VAT was incubated with biotinylated anti-mouse F4/80 antibody (eBioscience, 13-4801-85) in isolation buffer (PBS, 0.1% BSA, 2mM EDTA, pH 7.4). Cells were washed and incubated with prewashed magnetic beads (Dynabeads Biotin binder, Life Technologies, 11047) to isolate the F4/80+ fraction.

### BMDM culture.

Mouse femurs and tibias were collected in complete media containing RPMI (Thermo Fischer Scientific), 10% FBS (Omega Scientific), and 1% antibiotics/antimycotic (Thermo Fischer Scientific). Bone marrow was flushed into new complete media by a needle and syringe. RBCs were lysed by ACK lysis buffer (Quality Biological) followed by neutralization with collection media. The collected cells were seeded in 6-well plates and differentiated into macrophages using 10 ng/mL M-CSF (R&D) and L929 (ATCC) conditioned media. Nonadherent cells were harvested on day 7, and seeded as 1 × 10^6^ cell/well in a 24-well plate. Cells were treated with recombinant SPARC protein (20 μg/mL) (PeproTech) for 24 hours or the time indicated in Figure 8C on day 8. For the siRNA experiment, BMDMs in Opti-MEM media (Thermo Fischer Scientific) were transfected with specific siRNA against *Jnk1*, *Jnk2*, and *Stat1* (4390771-s77119, s77122, s74444; Thermo Fischer Scientific) along with negative control (4390843; Thermo Fischer Scientific) using Lipofectamine RNAiMAX following manufacturer’s instruction. Media was replaced with complete RPMI after 4 hours and incubated for 48 hours total. Then, cells were treated with recombinant SPARC protein (20 μg/mL) (PeproTech) for 24 hours. For inflammasome activation, BMDMs were treated with 1 μg/mL LPS for 4 hours as a priming, followed by 5 mM ATP treatment for 1 hour. SPARC recombinant protein (1, 5, and 20 μg/mL) was pretreated for 24 hours and cotreated with LPS, or ATP.

### Real-time reverse-transcription PCR.

RNA was extracted and purified using RNeasy Plus mini and micro kit (Qiagen) according to manufacturer’s instructions. RNA concentration was measured, and cDNA synthesis was performed using iScript cDNA synthesis kit (Bio-Rad). Real time qPCR was performed with cDNA, gene specific primers, and Power SYBR Green detection reagent (Thermo Fischer Scientific) in the LightCycler 480 II (Roche). Measured values from specific genes were analyzed by ΔΔCt method and normalized with *Gapdh* gene as an endogenous control. Gene primer sequences are in Supplemental Table 2.

### Western blotting.

Tissues were harvested followed by snap freezing in liquid nitrogen, then homogenized in RIPA buffer with protease inhibitors. Cell lysates were prepared by harvesting in RIPA buffer with protease inhibitors. Equal amounts of protein were run on SDS-PAGE gels after quantification of protein concentration using the DC protein assay (Bio-Rad) and transferred to nitrocellulose membrane. Primary antibodies and appropriate secondary antibodies were used to probe blots. The bands were detected by chemiluminescent visualization (PI32209; Pierce). The following primary antibodies were used for experiments. Antibodies to SPARC (1:1,000; AF942, R&D), β-actin (1:1,000; 4967L, Cell Signaling), p-AKT (1:1,000; 9271S, Cell Signaling), AKT (1:1,000; 9272S, Cell Signaling), PPARγ (1:200; sc-7273, Santa Cruz), p-HSL (S563) (1:1,000; 4139, Cell Signaling), HSL (1:1,000; 4107, Cell Signaling), IL-1β (1:1,000; GTX74034, Genetex), p-JNK (1:1,000; 4668S, Cell Signaling), JNK (1:1,000; 9252S, Cell Signaling), p-p38 MAPK (1:1,000; 4511S, Cell Signaling), p38 MAPK (1:1,000; 8690S, Cell Signaling), NF-kB p65 (1:1,000; 8242S, Cell Signaling), p-NF-kB p65 (1:1,000; 3033S, Cell Signaling), STAT1 (1:1,000; 9172S, Cell Signaling), and Caspase-1 (1:250; a gift from Genentech) were used. ImageJ was used for densitometry analysis.

### GTT/ITT.

For the glucose tolerance test (GTT), mice were fasted 14 hours and 10% glucose solution (Sigma Aldrich) was delivered by i.p. injection based on body weight (0.4 g/kg). For the insulin tolerance test (ITT), mice were fasted for 4 hours and insulin was given by i.p. injection (0.8 U/kg). For both GTT and ITT, the level of blood glucose was measured by handheld glucometer (Breeze, Bayer Health Care) at baseline and multiple time points.

### MRI.

The parameters of body composition were measured in vivo by MRI (EchoMRI; Echo Medical Systems). The amount of fat mass, lean mass, and free water were measured by the analysis. For the analysis, each mouse was placed in an acrylic tube with breathing holes and the tube was inserted in the MRI machine. The analysis per mouse takes approximately 90 seconds and automatically calculated numerical results were analyzed.

### Metabolic cage.

The EE, activity, food intake, and water consumption of mice were monitored using the TSE PhenoMaster System (V3.0.3) Indirect Calorimetry System. Each mouse was located in individual chambers for the time indicated in Figure 5 and Supplemental Figure 4 and the parameters were measured every 30 minutes. EE was calculated based on the oxygen consumption (O_2_) and carbon dioxide production (CO_2_). The EE was unnormalized or normalized by body weight to be used for analysis. Mouse activity was detected by infrared sensors, and food intake and water consumption were measured via weight sensors on food and water dispensers located in the cage.

### Lipolysis.

For ex vivo lipolysis assay, mice were either fed or fasted for 24 hours until they were euthanized for collecting adipose tissues. 15 mg of VAT and SAT was incubated in 100 μL lipolysis buffer (Krebs buffer with 0.1% glucose and 3.5% fatty-acid free BSA; Sigma-Aldrich) in a 96-well plate for 2 hours at 37°C at 450 rpm (or 0.34*g*) on the thermomixer (Eppendorf). Tissues from fasted mice were incubated without stimulating reagents, while tissues from fed mice were incubated with stimulating reagents such as with 1 μM noradrenaline or 2 mM isopreterenol. With supernatants from lipolysis assay, glycerol assays (Sigma-Aldrich) and FFA assays (WAKO) was performed following manufacturer’s instruction. For SPARC treatment, adipose tissues from fed mice were preincubated with 20 μg/mL SPARC for 24 hours before lipolysis assay.

### RNA-Seq analysis.

RNA that passed quality checks was used for RNA-Seq library preparation at Yale Center for Genome Analysis following manufacturer’s instruction (Illumina). HiSeq 2500 was used for the library sequencing. The sequencing quality of raw reads was assessed with FastQC (v0.11.3). Raw reads were mapped to hg38 human genome (GENCODE v29) (50) using STAR aligner (v2.5.3a) with the following options: --outFilterMultimapNmax 15 --outFilterMismatchNmax 6 --outReadsUnmapped Fastx --outSAMstrandField intronMotif --outSAMtype BAM SortedByCoordinate. Quality control of the mapped reads was done using Picard tools (v2.18.4). Quantification was done using htseq-count function from HTSeq framework (v0.9.1) (51): htseq-count -f bam -r pos -s no -t exon. To visualize expression levels by heatmaps, count data were first normalized using VST method from DESeq2 R package (v1.24.0) (52), and then donor effect was removed from normalized data using ComBat function from SVA R package (v3.32.1). Differential expression analysis was done using DESeq function from DESeq2 with default settings and the following design: gene ~ donor + timepoint. The significance threshold was set to FDR < 5%. rpkm function from edgeR package (v3.26.8) (53) was used to calculate RPKM values. Number of coding bases of each gene was used for normalization.

### Data availability.

The bulk RNA-Seq data is available at https://www.ncbi.nlm.nih.gov/sra/PRJNA1018321 Values for all data points in graphs are reported in the Supporting Data Values file.

### Statistics.

2-tailed Student’s *t* tests were used for calculating statistical significance. For correlation analysis between human phenotypes and RNA-Seq result, Pearson correlation analysis was used. Significance was established as *P* < 0.05. If needed to determine exclusion criteria, outliers were statistically defined by GraphPad Prism (GraphPad Software, San Diego, California, USA), then excluded from data analysis. A 95% CI was used for all statistical tests, and data was assumed to be normally distributed. Determination of sample size in each experiment was by previously published experiments. Numbers of biological replication and the independent experiment repeat were indicated in each figure legend corresponding to each experiment. Data were shown as mean ± SEM. GraphPad Prism was used for all statistical tests for analysis of experimental results.

### Study approval.

All human studies were performed under protocol approved by the Pennington IRB with informed consent from participants. All mouse experiments and animal use were conducted in compliance with the NIH Guide for the Care and Use of Laboratory Animals and were approved by the IACUC at Yale University and Pennington Biomedical Research Center.

## Author contributions

SR performed experiments, data analysis, and prepared the manuscript. OS and YHY performed RNA-seq of CALERIE-II samples and assisted in mouse phenotyping. AHL performed experiments on mice with protein-restricted diets. SC provided human adipocytes with diet restriction. SS and IS performed RNA-seq analysis. CM provided mouse adipose tissue samples from the protein-restriction diet model. ER and SRS were involved in the design of parent CALERIE-II trial, participant recruitment, and execution of study at PBRC-Baton Rouge clinical site. SRS also performed the biopsies of human adipose tissue. MNA supervised RNA-Seq analysis and assisted in transcriptomic data interpretation. All authors participated in manuscript preparation. VDD conceived the project, helped with data interpretation, analysis, and wrote the manuscript.

## Supplementary Material

Supplemental data

Supporting data values

## Version 1 10/02/2023

Electronic publication
