# Supplementary material for: Reduction of SPARC protects mice against NLRP3 inflammasome activation and obesity
Source: J Clin Invest. 2023 Oct 2;133(19):e169173. doi: 10.1172/JCI169173 (PMC10541189; doi:10.1172/JCI169173)
Supplement: Supplemental data [file jci-133-169173-s111.pdf]

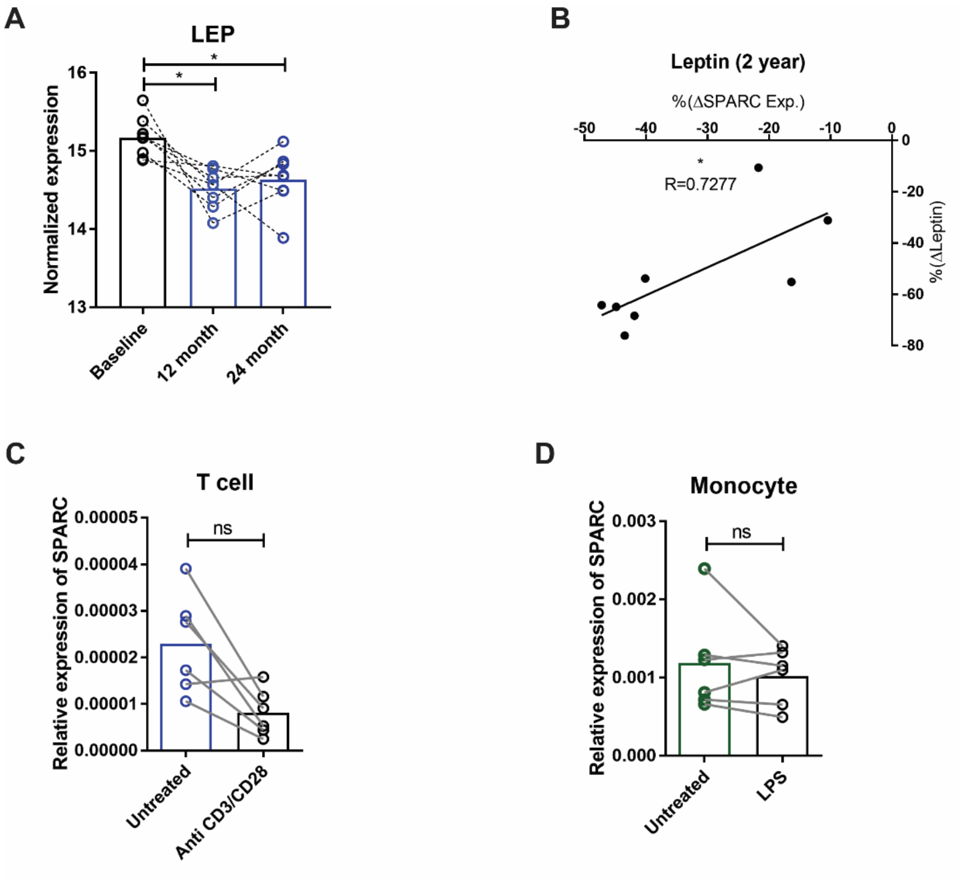

Supplemental Figure 1. SPARC is an adipokine identified in human CR study. (A) Normalized expression level of *LEP* gene in RNA-sequencing with human adipose tissue at baseline, 1 year and 2 years of CR (n=8). The adjusted p-value is indicated. **(B)** Correlation analysis of *SPARC* gene expression change and circulating Leptin level with 2 years of CR. **(C, D)** *SPARC* gene expression in human T cells with or without activation by anti CD3/CD28 **(C)** and human monocyte treated with or without LPS **(D).** Error bars represent the mean ± S.E.M. Two-tailed unpaired t-tests and Pearson correlation analysis test were performed for statistical analysis. ns indicates not significant; * $P < 0.05$.

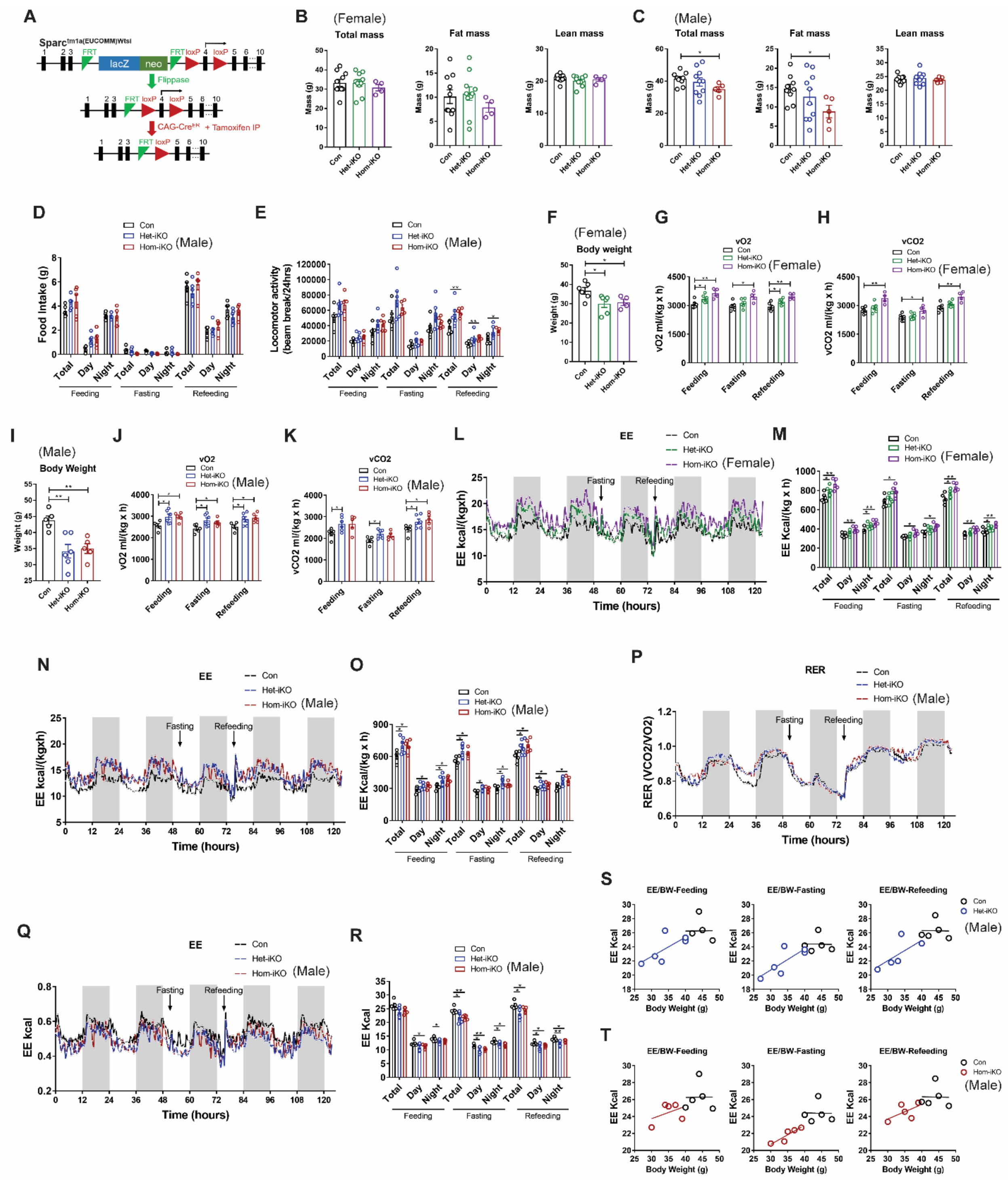

Supplemental Figure 2. SPARC global inducible KO in mice lowers adiposity and alters energy expenditure. (A) Schematic overview of global inducible SPARC KO generation using Flippase (Flp) and CAG-Cre^{ER} with tamoxifen intraperitoneal (IP) injection. **(B, C)** Body

composition of 15-month old female **(B)** and male (**C**) Con, Het-iKO, and Hom-iKO mice (n=10, 10, 4 and n=10, 10, 5). **(D, E)** Analysis results of food intake **(D)**, and locomotive activity **(E)** of the male mice. **(F-P)** Body weight **(F, I)** normalized vO2 of female and male mice **(G, J)**, vCO2 of female and male mice **(H, K)**, energy expenditure of female and male mice **(L, N)**, energy expenditure with time scale in female and male mice **(M, O)**, and RER in male mice **(P)**. **(Q, R)** Unnormalized EE by metabolic cage analysis of 15-month old male Con, Het-iKO, and Hom-iKO mice (n=5, 6, 5). **(S, T)** Comparison of linear regression analyses about unnormalized energy expenditure (EE) and body mass between male Con and Het-iKO mice (n=5, 6); ANCOVA **(S)** and between male Con and Hom-iKO mice (n=5, 5) **(T)**. Error bars represent the mean ± S.E.M. Two-tailed unpaired t-tests were performed for statistical analysis. * $P < 0.05$; ** $P < 0.01$.

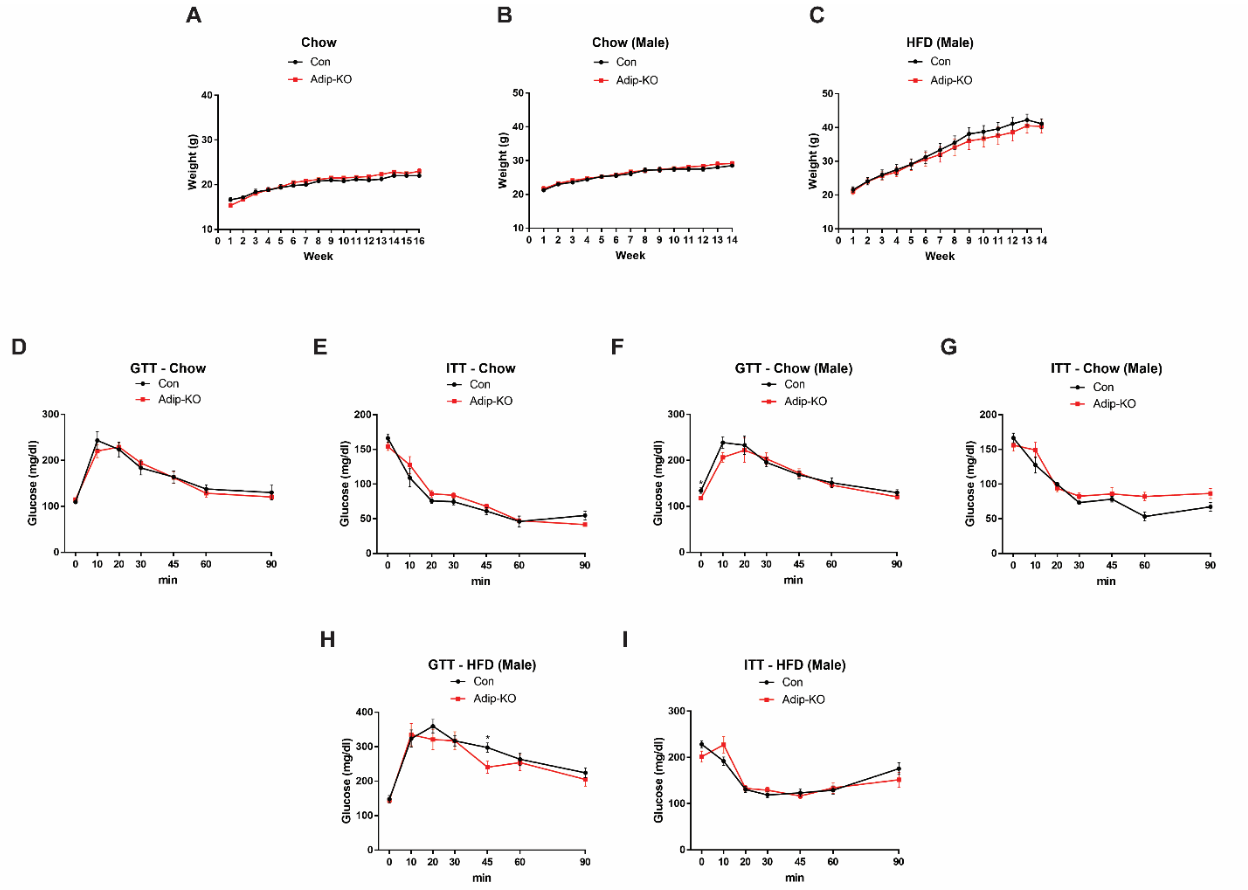

Supplemental Figure 3. Adipocyte-SPARC depletion in HFD-induced obesity protects against metabolic dysregulation. (A) Weight change of female Con and Adip-KO mice during 16 weeks of chow diet (n=5, 6). **(B, C)** Weight change of male Con and Adip-KO mice during 16 weeks of chow diet (n=7, 7) **(B)** and HFD (n=8, 7) **(C)**. **(D, E)** GTT and ITT of female Con and Adip-KO mice after 16 weeks of chow diet (n=4, 6). **(F-I)** GTT and ITT of male Con and Adip-KO mice after 16 weeks of chow diet **(F, G)** (n=6, 7, n=4, 4) and HFD **(H, I)** (n=8, 6). Error bars represent the mean ± S.E.M. Two-tailed unpaired t-tests were performed for statistical analysis. * $P < 0.05$.

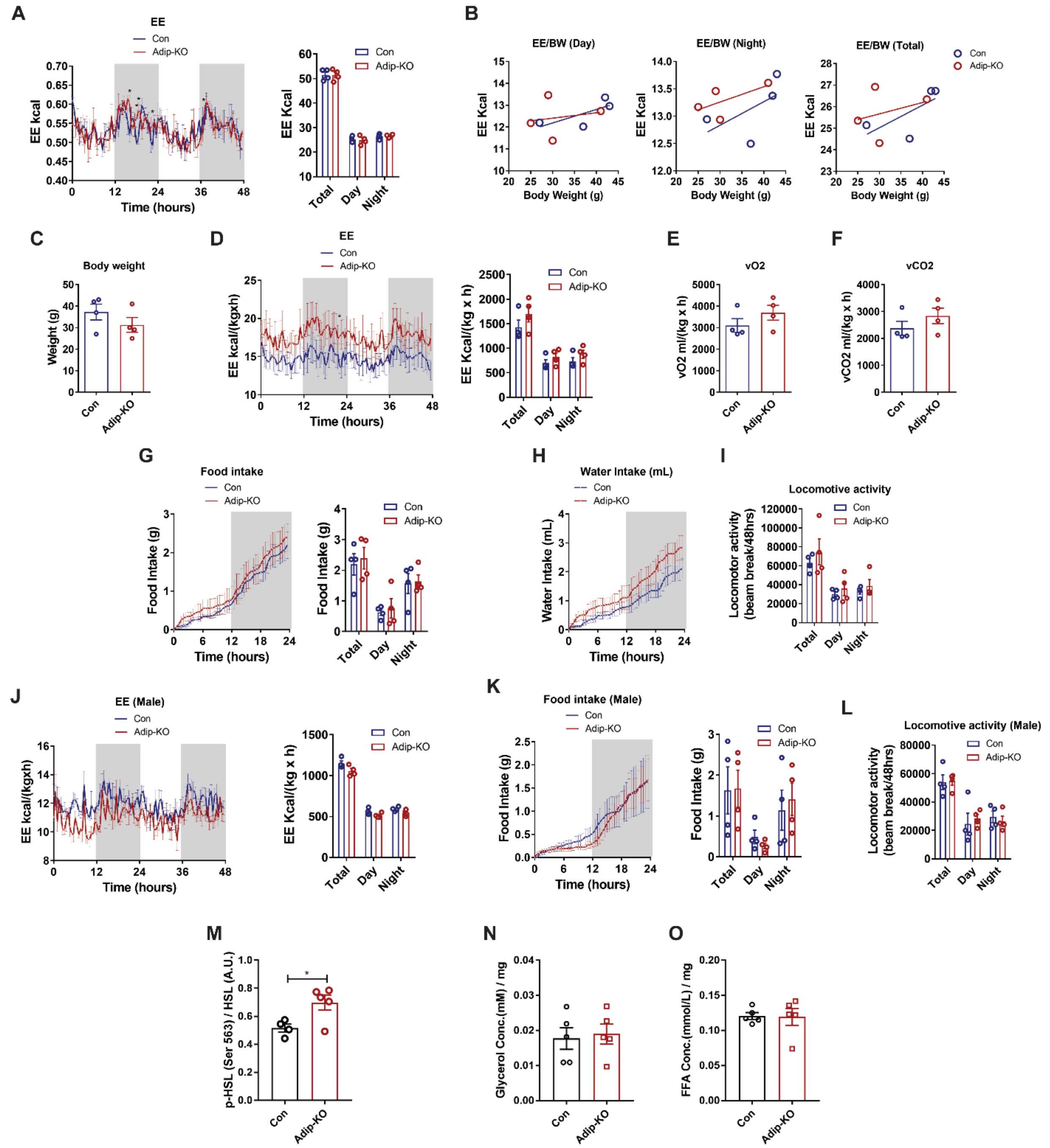

Supplemental Figure 4. Adipocyte produced SPARC regulates lipolysis and energy expenditure. (A, B) Unnormalized EE analysis measured by indirect calorimetry in Con and Adip-KO mice fed HFD (n=4, 4) **(A)** and comparison of linear regression lines at day, night, and total **(B)**. **(C-F)** Body weight **(C)** normalized metabolic cage detected parameters of female Con

and Adip-KO mice with HFD (n=4, 4). EE **(D)**, vO2 **(E)**, and vCO2 **(F)** analysis results were indicated. **(G-I)** food intake **(G)**, water intake **(H)** and locomotive activity **(I)** analysis results were indicated. **(J-L)** Parameters detected in metabolic cage experiment of male Con and Adip-KO mice with HFD (n=4, 4). Normalized EE **(J)**, food intake **(K)**, and locomotive activity **(L)** analysis results were indicated. **(M)** Quantification of lipolysis signaling in adipose tissue explant (SAT) from Con and Adip-KO mice after 24 hr of fasting (n=4, 5). **(N, O)** Glycerol **(N)** and FFA **(O)** assay with explant of VAT from male Con and Adip-KO mice (n=5, 5). Error bars represent the mean ± S.E.M. Two-tailed unpaired t-tests were performed for statistical analysis. * $P < 0.05$.

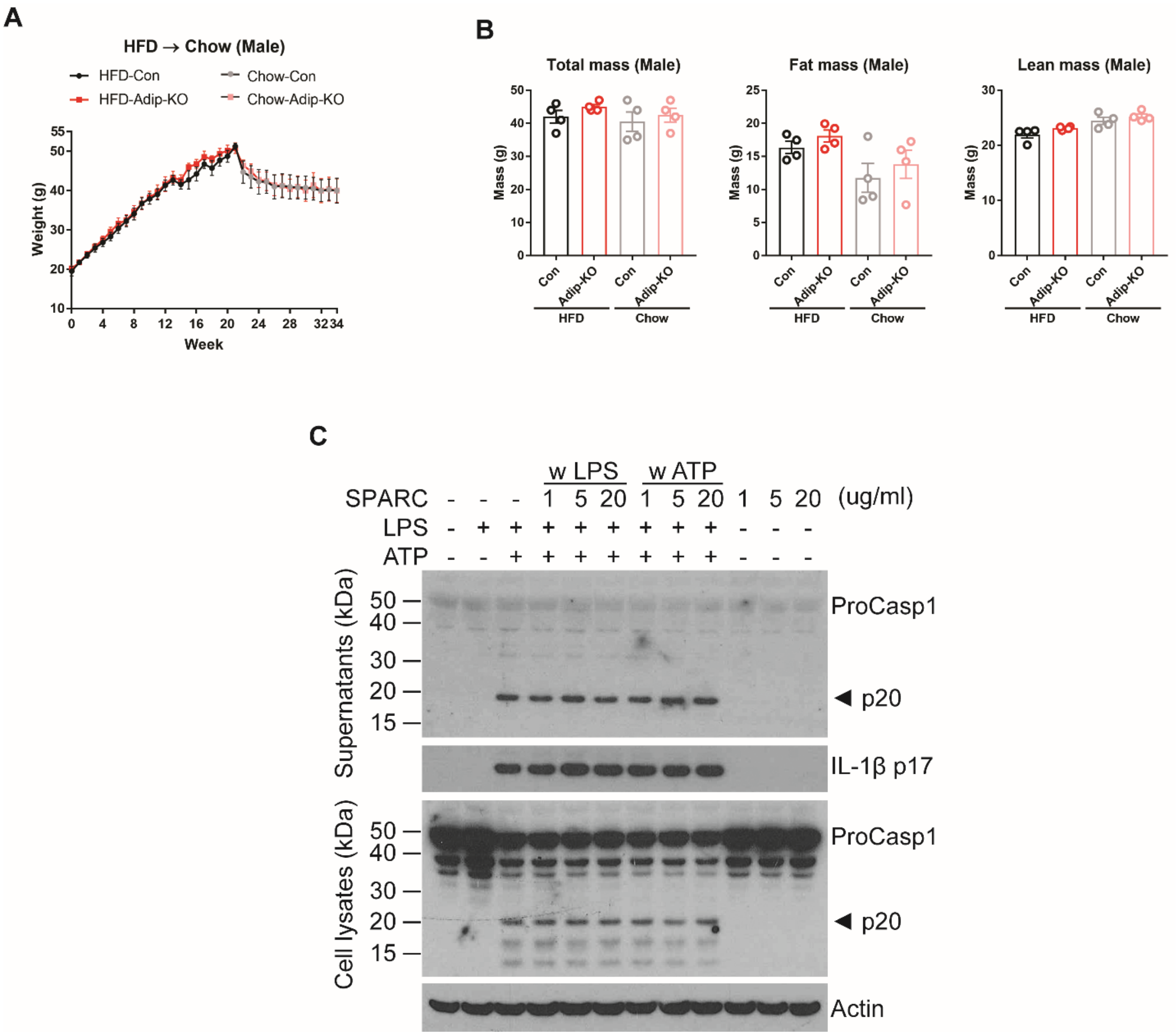

Supplemental Figure 5. Macrophage inflammation is regulated by SPARC level. (A) Weight change of male Con and Adip-KO mice with 21 weeks of HFD followed by 13 weeks of chow diet (n=4, 5). **(B)** Body composition analysis of male Con and Adip-KO mice before and after diet change from HFD to chow diet. **(C)** Western blot analysis in conditions of SPARC (1, 5, 20 μg/ml) alone, co-treatment with LPS (w LPS) for 5 hr, or co-treatment with ATP (w ATP) for 1 hr to measure inflammsome activation. Activation was detected by cleaved form of caspase-1 (Casp1, p20) and IL-1β (p17) in supernatant (upper two) and cell lysate (lower two) of

BMDMs. ProCasp1indicates the pro-form of Caspase-1. Error bars represent the mean ± S.E.M. Two-tailed unpaired t-tests were performed for statistical analysis.

Supplemental Table 1. Information of study participants who provided adipose tissues for RNA-sequencing.

Subject ID	A00-01-0004	A00-01-0039	A00-01-0044	A00-01-0049	A00-01-0051	A00-02-0117	A00-03-0024	A00-03-0109	Mean	SD
Sex	Male	Female	Female	Male	Female	Female	Female	Female	-	-
Age	41.6	45.7	44.4	26.3	32.6	30.8	34.8	32.1	36.0	7.0
Body mass index	25.8	25.4	24.2	24.9	22.9	28.4	24.0	26.8	25.3	1.7
Weight change % (1 year)	13.76	15.51	13.33	9.45	13.82	8.62	7.29	15.89	12.2	3.3
Weight change % (2 year)	10.25	14.08	14.25	10.37	14.98	4.54	5.45	13.12	10.9	4.0

Supplemental Table 2. Primer information for qPCR.

Gene	Direction	5'-3'
Sparc	Forward	TGCAAATACATCGCCCCCTG
	Reverse	ACATTTTTGAGCCAGTCACGC
Il1b	Forward	GGTCAAAGGTTTGGAAGCAG
	Reverse	TGTGAAATGCCACCTTTTGA
Tnf	Forward	TCTCAGCCTCTTCTCATT
	Reverse	AGAACTGATGAGAGGGAG
Il6	Forward	AGACAAAGCCAGAGTCCTTCAGAG
	Reverse	TTGGTCCTTAGCCACTCCTTCTGT
Nos2	Forward	CCCTCCTGATCTTGTGTTGG
	Reverse	GGCAGTGCATACCACTTCAA
Casp1	Forward	GGACCCTCAAGTTTTGCCCT
	Reverse	AGACGTGTACGAGTGGTTGT
Nlrp3	Forward	GCTAAGAAGGACCAGCCAGA
	Reverse	CAGCAAACCCATCCACTCTT
Gapdh	Forward	TCAACAGCAACTCCCACTCTTCCA
	Reverse	ACCCTGTTGCTGTAGCCGTATTCA
